# Detecting context dependence in the expression of life history trade‐offs

**DOI:** 10.1111/1365-2656.14173

**Published:** 2024-09-02

**Authors:** Louis Bliard, Jordan S. Martin, Maria Paniw, Daniel T. Blumstein, Julien G. A. Martin, Josephine M. Pemberton, Daniel H. Nussey, Dylan Z. Childs, Arpat Ozgul

**Affiliations:** ^1^ Department of Evolutionary Biology and Environmental Studies Zurich University Zurich Switzerland; ^2^ Institute of Evolutionary Medicine Zurich University Zurich Switzerland; ^3^ Department of Conservation Biology Estación Biológica de Doñana (EBD‐CSIC) Seville Spain; ^4^ Department of Ecology and Evolutionary Biology University of California Los Angeles California Los Angeles USA; ^5^ The Rocky Mountain Biological Laboratory Crested Butte Colorado USA; ^6^ Department of Biology University of Ottawa Ottawa Ontario Canada; ^7^ Institute of Ecology and Evolution University of Edinburgh Edinburgh UK; ^8^ Department of Animal and Plant Sciences University of Sheffield Sheffield UK

**Keywords:** covariance reaction norm, demography, heterogeneity, life‐history, mixed effects, multivariate model, phenotypic correlation, trade‐off

## Abstract

1. Life history trade‐offs are one of the central tenets of evolutionary demography. Trade‐offs, depicting negative covariances between individuals' life history traits, can arise from genetic constraints, or from a finite amount of resources that each individual has to allocate in a zero‐sum game between somatic and reproductive functions. While theory predicts that trade‐offs are ubiquitous, empirical studies have often failed to detect such negative covariances in wild populations.
2. One way to improve the detection of trade‐offs is by accounting for the environmental context, as trade‐off expression may depend on environmental conditions. However, current methodologies usually search for fixed covariances between traits, thereby ignoring their context dependence.
3. Here, we present a hierarchical multivariate ‘covariance reaction norm’ model, adapted from Martin (2023), to help detect context dependence in the expression of life‐history trade‐offs using demographic data. The method allows continuous variation in the phenotypic correlation between traits. We validate the model on simulated data for both intraindividual and intergenerational trade‐offs.
4. We then apply it to empirical datasets of yellow‐bellied marmots (*Marmota flaviventer*) and Soay sheep (*Ovis aries*) as a proof‐of‐concept showing that new insights can be gained by applying our methodology, such as detecting trade‐offs only in specific environments.
5. We discuss its potential for application to many of the existing long‐term demographic datasets and how it could improve our understanding of trade‐off expression in particular, and life history theory in general.

## Graphical abstract

This proof‐of‐concept paper highlights that the context dependence of trade‐offs can be successfully detected, hence potentially improving our understanding of life‐history theory. Photo credit: Arpat Ozgul. Sheep illustration from Phylopic by Gabriela Palomo‐Munoz.

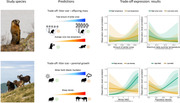

## 1 INTRODUCTION

Demographic trade‐offs, which are characterised as negative covariances between fitness components such as somatic or reproductive traits, are central to life history theory (Stearns, 1989), and are thought to constrain and organise much of the life history diversity that exists (Bielby et al., 2007; Healy et al., 2019; Salguero‐Gómez et al., 2016; Stearns, 1984). They originate from the basic fact that the total amount of resources or energy acquired by any one individual is limited, and has to be shared among several of the individual's fitness‐related traits. In such a zero‐sum game and in the absence of change in the total amount of resources acquired, any increase in the allocation of resources towards a specific fitness component will have to be at the expense of another fitness component. While trade‐offs stem from individual processes, these covariances can scale up to different levels of organisation (Agrawal, 2020; Bliard, Paniw, et al., 2024). If trade‐offs did not exist, selection would maximise all fitness‐related traits simultaneously and would lead to the impossible “darwinian demons” (Law, 1979). Therefore, trade‐offs should be faced by all organisms and are, in theory, ubiquitous (Stearns, 1989, 1992; Williams, 1966). They can come in several forms (Stearns, 1989), being either intraindividual (traits involved relate to the fitness of the same individual) or intergenerational (traits involved relate to the fitness of a parent‐offspring pair; e.g. offspring quantity‐quality trade‐off). Despite their expected universality and being sought‐after by evolutionary ecologists and biodemographers alike, life‐history trade‐offs have been surprisingly hard to detect in wild populations (Chang et al., 2023; Metcalf, 2016; van Noordwijk & de Jong, 1986), with successful probes too often confined to experimental approaches.

Several reasons could explain why trade‐offs are hard to detect in wild populations. First, we often expect traits to covary in a simple bivariate manner following the Y‐model of resource allocation, where any resources diverted from a trait will be allocated to the other one (de Jong & van Noordwijk, 1992). Thus, while we are often analysing a single pair of traits at a time, trade‐off structures are often more complex. For instance, many more than two traits are likely to be involved in the resource allocation process (Cressler et al., 2017; de Jong, 1993; Pease & Bull, 1988), sometimes leading to complex hierarchical allocation trees, potentially resulting in some pairs of traits not covarying negatively (Gascoigne et al., 2022). Second, life history traits can covary at different levels. While trade‐offs result from individuals' resource allocation processes, biodemographers often study trade‐offs as the temporal correlations among demographic rates at the population level (Compagnoni et al., 2016; Fay et al., 2020; Fay, Hamel, et al., 2022; van Tienderen, 1995). Trade‐offs can occasionally scale up to cause negative temporal covariances at the population level (van Tienderen, 1995), but in most cases these covariances are the results of environmental stochasticity and demographic reaction norms to shared ecological drivers (Fay, Hamel, et al., 2022; Knops et al., 2007; Paniw et al., 2020). Third, even though trade‐offs might be present, individual heterogeneity can mask their presence among individuals. This specific ecological version of Simpson's paradox (Simpson, 1951) has been demonstrated by van Noordwijk and de Jong (1986): when the among‐individual variance in resource acquisition is greater than the among‐individual variance in resource allocation, the trade‐off is not expressed among individuals—even though it is theoretically present within individuals. In addition, expression of a trade‐off among individuals can also be influenced if the allocation and acquisition processes are not independent (Descamps et al., 2016; Fischer et al., 2009; Robinson & Beckerman, 2013). Altogether, this makes the detection of trade‐offs in wild populations difficult.

How much individuals vary in acquisition and allocation of resources determines if a trade‐off is detected among individuals (Metcalf, 2016; Reznick et al., 2000; van Noordwijk & de Jong, 1986). Part of this variance might be fixed, stemming from genetic, developmental, or consistent behavioural differences that constrain how much resources are acquired and allocated to somatic versus reproductive functions (Réale et al., 2007; Wilson & Nussey, 2010). The remaining variance is likely to be plastic (Spigler & Woodard, 2019), where acquisition versus allocation likely depends on the environmental context (Cohen et al., 2020; Sgrò & Hoffmann, 2004; Stearns et al., 1991). For instance, in several species, no trade‐offs were found among captive animals fed ad libitum (Kengeri et al., 2013; Landes et al., 2019; Ricklefs & Cadena, 2007). Similarly, controlled laboratory experiments on several species have shown that trade‐offs detection and strength were dependent on resource abundance (Gebhardt & Stearns, 1988; Messina & Fry, 2003; Messina & Slade, 1999; Spigler & Woodard, 2019). However, despite evidence that trade‐off expression depends on the environmental context, statistical methods to detect this context dependence in wild populations have, to date, rarely been applied.

Multivariate models are commonly employed to detect trade‐offs in wild populations (Cam et al., 2002, 2013; Fay, Authier, et al., 2022; Hamel et al., 2018; Paterson et al., 2018). In quantitative genetics, such models allow for the simultaneous analysis of multiple dependent variables like fecundity, growth and survival (Kruuk et al., 2008; Wilson, Réale, et al., 2010). These variables each have their own predictors, and the models estimate the correlated residual variances unaccounted for by the primary predictors. These models can be used to study residual correlations between traits at different levels, such as among‐year correlation and among‐individual correlation. For example, after accounting for primary predictors, such models quantify whether years with high survival in a population are also years with high recruitment; or whether individuals with higher fecundity have lower or higher growth rates. However, these correlations among residual variances are estimated as fixed. Estimating fixed correlations might not necessarily be problematic in the case of experimental work, in which environmental conditions can be held constant within each treatment. However, wild populations are unlikely to experience fixed conditions, as the environmental context will vary in a continuous fashion, hence influencing the expression of trade‐offs. Therefore, there is a need to analyse and predict continuous variation of phenotypic correlations.

Here, we repurpose a hierarchical multivariate ‘covariance reaction norm’ (hereafter CRN) model recently developed by Martin (2023), which allows the incorporation of continuous predictors directly on the covariance matrix, for application to sampling designs typical in population ecology, enabling the study of the context‐dependent expression of trade‐offs. As a proof‐of‐concept, we first validate this model on two simulated datasets, respectively focusing on an intergenerational trade‐off and an intraindividual trade‐off. We then apply our model on two empirical datasets of wild populations of yellow‐bellied marmots *Marmota flaviventer* and Soay sheep *Ovis aries*. Prior studies have explored trade‐offs between vital rates in both species (Kroeger et al., 2020; Tavecchia et al., 2005). For instance, in yellow‐bellied marmots, a quality‐quantity trade‐off in offspring has been observed for older mothers. In Soay sheep, the costs of reproduction have been particularly evident for breeding ewes in high‐density populations or following harsh winters. However, the environmental context‐dependence of these trade‐offs has yet to be studied explicitly. In the marmots, which inhabit high‐altitude, highly seasonal environments, and the sheep, which face severe winter storms and fluctuating population densities, we hypothesise that trade‐offs are more likely to manifest under unfavourable ecological conditions (Cohen et al., 2020; Sgrò & Hoffmann, 2004).

## 2 METHODS

### 2.1 The model

In this study, we employ a newly introduced CRN model (Martin, 2023), which has been developed as a quantitative genetic model to predict continuous changes in trait associations when either genetic data or repeated individual measurements are available for all phenotypes of interest. A key assumption of multivariate models thus far has been that phenotypic correlations caused by trade‐offs are fixed through time or space (Cam et al., 2002; Hamel et al., 2018). The CRN approach provides a solution to this general challenge, by allowing for phenotypic covariances to vary in response to variation in the environment, for example, estimating under which conditions among‐individual variance in resources allocation is larger than among‐individual variance in acquisition (van Noordwijk & de Jong, 1986). In the present study, we extend application of this general CRN approach to the detection of context‐dependent trade‐offs (here defined as among‐individual correlations even though both are not always equivalent) between life history traits, with special consideration to sampling conditions typical of long‐term field research in population ecology. Specifically, we examine the use of bivariate CRN models to test for the presence of phenotypic trade‐offs when repeated individual measurements are lacking in a given environmental context (e.g. during a specific sampling event such as a breeding season or a year). These are typical situations in field research that motivate further development of the quantitative genetic models proposed by Martin (2023).

Before delving into the specifics of the model, note that in all the following models presented, measurements of the same individuals observed in different contexts are considered independent (see Section S1 for more details). This necessary simplification has potential consequences when searching for the phenotypic manifestation of trade‐offs, as fixed heterogeneity across ecological contexts cannot be properly disentangled from context‐dependent heterogeneity, which might lead to issues especially in long‐lived species that are observed across many different contexts. Nonetheless, this simplification does not impede our ability to detect context‐dependence of among‐individual correlations (Section S1). Consider a CRN model investigating how environmental contexts C and individual factors affect the phenotypic means of $\beta_{\mu 1}$ and $\beta_{\mu 2}$ and among‐individual correlations $\beta_{r}$ between two Gaussian life history trait measures **z**_**1**_ and **z**_**2**_ with repeated individual measurements in each environmental context. **X**_**1**_ and **X**_**2**_ are N × P matrices of *N* measurements of *P* predictors. We begin by focusing on linear models to simplify notation and aid comprehension, with generalised models for non‐Gaussian distributions discussed further below. Following Martin (2023) in the absence of genetic data, our bivariate phenotypic model is given by

(1.1) $$\begin{array}{c} z_{1}=X_{1}\beta_{\mu 1}+{\mathrm{W\alpha}}_{1\left(C\right)}+\epsilon_{1\left(C\right)}\\ z_{2}=X_{2}\beta_{\mu 2}+{\mathrm{W\alpha}}_{2\left(C\right)}+\epsilon_{2\left(C\right)}\\ \left[\alpha_{1\left(C\right)},\alpha_{2\left(C\right)}\right]\sim N\left(0,P_{\left(C\right)}\right)\\ \left[\epsilon_{1},\epsilon_{2}\right]\sim N\left(0,\sum_{\left(C\right)}\right) \end{array}$$

Trait values are expressed as a function of the average effects 𝜷_**𝝁1**_ and 𝜷_**𝝁2**_ of **X**_**1**_ and **X**_**2**_ on each phenotype, as well as among‐individual effects 𝜶_**1(C)**_ and 𝜶_**2(C)**_ that are repeatable across measurements and within‐individual effects 𝜺_**1(C)**_ and 𝜺_**1(C)**_ that are variable across measurements. The model matrix **W** (an N × J matrix for *J* subjects) structures the among‐individual effects 𝜶_**(C)**_ across repeated measurements. (Co)variances between independent among‐ and within‐individual effects are respectively described by **P** and **𝚺** covariance matrices. To detect context‐dependent trade‐off expression, we use environmental information in **X**_**3**_ (an C × P matrix of *C* environmental contexts of *P* predictors) to predict the among‐individual trait covariance matrix **P**_**(C)**_,

(1.2) $$\begin{array}{c} P_{\left(C\right)}=\left[\begin{array}{cc} \sigma_{\alpha_{1}\left(C\right)}^{2} & r_{\alpha \left(C\right)}\sigma_{\alpha_{1}\left(C\right)}\sigma_{\alpha_{2}\left(C\right)}\\ r_{\alpha \left(C\right)}\sigma_{\alpha_{2}\left(C\right)}\sigma_{\alpha_{1}\left(C\right)} & \sigma_{\alpha_{2}\left(C\right)}^{2} \end{array}\right]\\ \text{atanh}\left(r_{\alpha \left(C\right)}\right)=X_{3}\beta_{r} \end{array}$$

where the inverse hyperbolic tangent function atanh(*r*) = logit([*r* + 1]/2)/2 is used as a link function to model additive environmental effects 𝜷_**r**_ on the logit scale while retaining the [−1,1] scaling of the correlation coefficient *r*. This is akin to a logistic regression with bounds in [−1,1] instead of [0,1]. The same approach can be taken to describe changes in within‐individual variation across environmental contexts,

(1.3) $$\begin{array}{c} \sum_{\left(C\right)}=\left[\begin{array}{cc} \sigma_{\epsilon_{1}\left(C\right)}^{2} & r_{\epsilon \left(C\right)}\sigma_{\epsilon_{1}\left(C\right)}\sigma_{\epsilon_{2}\left(C\right)}\\ r_{\epsilon \left(C\right)}\sigma_{\epsilon_{2}\left(C\right)}\sigma_{\epsilon_{1}\left(C\right)} & \sigma_{\epsilon_{2}\left(C\right)}^{2} \end{array}\right]\\ \text{atanh}\left(r_{\epsilon \left(C\right)}\right)=X_{3}\beta_{r_{\epsilon }} \end{array}$$

Direct prediction of the transformed correlation coefficient is useful because we are principally interested in *r*_(*C*)_ as an indicator of putative trade‐offs, rather than the covariance $P_{1,2\left(C\right)}=r_{\left(C\right)}\sigma_{1}\sigma_{2}$ per se. Changes in the scale $\sigma_{1}\sigma_{2}$ of life history trait variation may occur independently of changes in positive or negative trait association among individuals, but these effects will be confounded together in the covariance $P_{1,2\left(C\right)}$. In contrast, the correlation coefficient *r*_(C)_ is standardised relative to the scale of each phenotype, providing a more robust quantity for directly predicting and comparing estimates of life history trade‐offs across phenotypes and species. Our model also assumes that phenotypic variances can vary across environmental contexts, but no predictions are made on this variation. Greater plasticity is instead expected in the strength of trade‐off expression caused by fluctuating environmental factors (e.g. environmental harshness, resource availability, local predator density). See Martin (2023) for further details on relaxing these assumptions to model environmental effects on among‐ and within‐individual variances.

#### 2.1.1 Non‐repeated measures

Estimating Equation 1 with empirical data requires multiple measurements of the same subjects to effectively partition trait correlations due to sources of among **P**_**(C)**_ and within‐individual ∑_**(C)**_ phenotypic variation, relative to a given window of sampling (i.e. a given environmental context C). Repeated individual measurements are often inconsistent or unavailable in a given environmental context (e.g. a single fecundity measurement for individuals in a given year) in long‐term field studies, which otherwise provide invaluable datasets for investigating context‐specific trade‐offs in the wild. Fortunately, we can still take advantage of long‐term environmental variation in such studies to detect variation in trade‐off expression without repeated measurements in a given environmental context. This requires simplifying the CRN model to predict observation‐level phenotypic associations across environmental contexts.

(2) $$\begin{array}{c} z_{1}=X_{1}\beta_{\mu 1}+o_{1\left(C\right)},\\ z_{2}=X_{2}\beta_{\mu 2}+o_{2\left(C\right)},\\ \left[o_{1\left(C\right)},o_{2\left(C\right)}\right]\sim N\left(0,P_{o\left(C\right)}\right),\\ P_{o\left(C\right)}=\left[\begin{array}{cc} \sigma_{o_{1}\left(C\right)}^{2} & r_{o\left(C\right)}\sigma_{o_{1}\left(C\right)}\sigma_{o_{2}\left(C\right)}\\ r_{o\left(C\right)}\sigma_{o_{2}\left(C\right)}\sigma_{o_{1}\left(C\right)} & \sigma_{o_{2}\left(C\right)}^{2} \end{array}\right],\\ \text{atanh}\left(r_{o\left(C\right)}\right)=X_{3}\beta_{r}. \end{array}$$

Here, the lack of repeated measurements mean that we cannot decompose the variance between among‐ and within‐individual variation. Therefore, ***o***_**1(C)**_ = 𝜶_**1(C)**_ + 𝜺_**1(C)**_ and ***o***_**2(C)**_ = 𝜶_**2(C)**_ + 𝜺_**2(C)**_ are observation‐level random effects aggregating variation due to among‐ and within‐individual differences across measurements, within a given environmental context defined by C (e.g. a given year, position in space, level of resource abundance). Note that the **W** matrix from Equation 1 is no longer necessary in Equation 2 in the absence of repeated measurements. As a consequence, we expect that the observation‐level correlation $r_{o\left(C\right)}$ between these random effects to reflect the combined effect of the among‐ and within‐individual correlations between life history traits, weighted by the geometric mean of their repeatability *R* (Dingemanse & Dochtermann, 2013; Searle, 1961).

(3) $$r_{o\left(C\right)}=r_{\alpha \left(C\right)}\sqrt{\frac{\sigma_{\alpha 1}^{2}}{\sigma_{z1}^{2}}\frac{\sigma_{\alpha 2}^{2}}{\sigma_{z2}^{2}}}+r_{\epsilon \left(C\right)}\sqrt{\frac{\sigma_{\epsilon 1}^{2}}{\sigma_{z1}^{2}}\frac{\sigma_{\epsilon 2}^{2}}{\sigma_{z2}^{2}}}=r_{\alpha \left(C\right)}\sqrt{R_{1}R_{2}}+r_{\epsilon \left(C\right)}\sqrt{\left(1-R_{1}\right)\left(1-R_{2}\right)}$$

**FIGURE 1:**
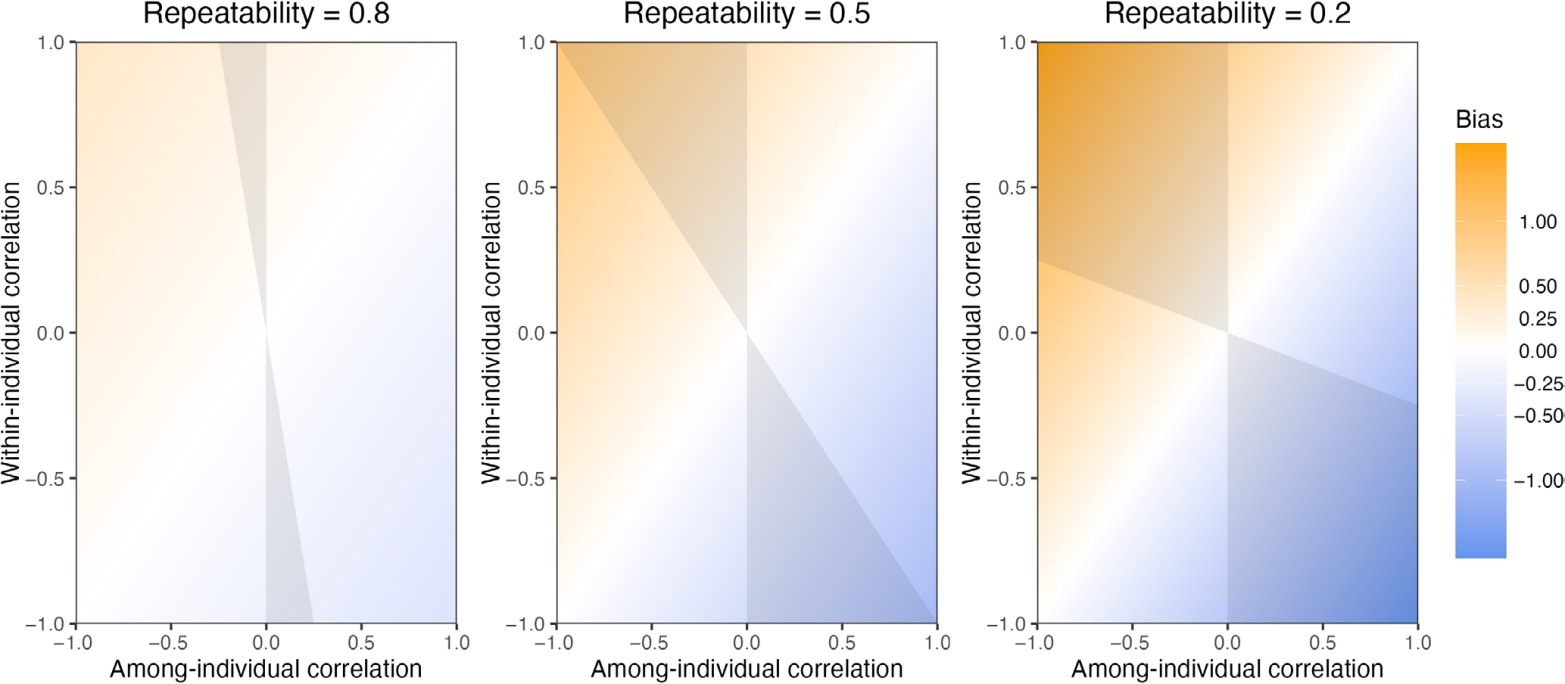
General relationships across correlations and repeatability ranges based on Equation 3 for a non‐repeated measures CRN (model of Equation 2), identifying the magnitude of correlation bias and the regions of sign bias. The bias is here defined as the difference between the observation‐level correlation and the among‐individual correlation, using the latter as a reference. Parameter spaces in grey represent the regions of sign bias, where the observation‐level correlation has a sign opposite to the among‐individual correlation. This highlights that the observation‐level correlation is mostly influenced by the among‐individual correlation for traits with high repeatability, while it is mostly influenced by the within‐individual correlation for traits with low repeatability.

Where phenotypic variances are adjusted for the mean effects of ***X***_***1***_𝜷_**𝝁1**_ and ***X***_***2***_𝜷_**𝝁2**_. We can see that inferences about among‐individual trade‐offs from the non‐repeated measures model (Equation 2) will be at greatest risk of bias when $\mathrm{sign}\left(r_{\alpha }\right)\neq \mathrm{sign}\left(r_{\epsilon }\right)$ and $\sqrt{R_{1}R_{2}}<<\sqrt{\left(1-R_{1}\right)\left(1-R_{2}\right)}$. Figure 1 shows these general relationships across correlation and repeatability ranges, identifying regions of sign bias. Fortunately, researchers will generally be able to judge their risk of inferential bias based on a priori knowledge about the repeatability of life history traits, which tends to be medium to high (Dingemanse et al., 2021). For example, observation‐level correlations of behavioural traits will tend to be dominated by within‐individual associations (Bell et al., 2009; Cauchoix et al., 2018; Holtmann et al., 2017), while morphological associations will tend to be dominated by among‐individual variation (Dingemanse et al., 2021). We reiterate that our models consider measurements of the same individuals observed in different contexts as independent (see Section S1). In addition, our model considers no measurement errors, as we are not able to disentangle it from true within‐individual variation using non‐repeated measures. Such considerations regarding trait repeatability and measurement error should be explicit when interpreting results without repeated measures.

#### 2.1.2 Hybrid scenarios

Variation in repeated sampling is also likely to occur across phenotypes due to factors such as difficulty of measurement and the rate of trait expression. While a single measure of age at first reproduction or fecundity in a given environmental context may be available per individual, multiple individual measures may be available for traits such as offspring quality. Such scenarios require a hybrid modelling approach. For example, consider a model with a single predictor for an intergenerational trade‐off between fecundity (e.g. clutch size) and offspring quality, but other traits could equally be studied. The model structure for offspring quality **z**_**1**_ (depicted as offspring body mass), a gaussian trait, is given by

(4.1) $$z_{1}=X_{1}\beta_{\mu 1}+{\mathrm{W\alpha}}_{1\left(C\right)}+\epsilon_{1\left(C\right)}$$

The linear predictor for ***z***_**1**_ (mass of an offspring of a given mother) in year C includes a year‐specific mother random effect 𝜶_**1(C)**_ and 𝜺_**1(C)**_ being the within‐brood/litter variance.

The model for fecundity ***z***_**2**_ follows the same basic structure, with a single fecundity measurement per female per year. We can use a Poisson distribution where we model the expected rate of offspring production using a log link function, but other distributions could equally be used,

(4.2) $$z_{2}=X_{2}\beta_{\mu 2}+o_{2\left(C\right)}$$

Without repeated measures, the random effect ***o***_**2(C)**_ is specified at the observation‐level, accounting for any overdispersion in the Poisson process across measurements of each female.

The context‐dependent trade‐off will be estimated between the among‐mother random effect in offspring quality and the observation‐level random effect in fecundity

(4.3) $$\begin{array}{c} \left[\alpha_{1\left(C\right)},o_{2\left(C\right)}\right]\sim N\left(0,P_{\left(C\right)}\right)\\ P_{\left(C\right)}=\left[\begin{array}{cc} \sigma_{\alpha_{1}\left(C\right)}^{2} & r_{\left(C\right)}\sigma_{\alpha_{1}\left(C\right)}\sigma_{o_{2}\left(C\right)}\\ r_{\left(C\right)}\sigma_{\alpha_{1}\left(C\right)}\sigma_{o_{2}\left(C\right)} & \sigma_{o_{2}\left(C\right)}^{2} \end{array}\right]\\ \text{atanh}\left(r_{\left(C\right)}\right)=X_{3}\beta_{r} \end{array}$$

Reducing Equation 3, the correlation $r_{C}$ between the individual‐ 𝜶_**1(C)**_ and observation‐level ***o***_**2(C)**_ effects will necessarily be proportional to the among‐individual correlation across life history traits,

(5) $$r_{o\left(C\right)}=r_{\alpha \left(C\right)}\sqrt{\frac{\sigma_{\alpha 2}^{2}}{\sigma_{z2}^{2}}}=r_{\alpha \left(C\right)}\sqrt{R_{2}}$$

Note that this method does not allow the inclusion of non‐continuous traits (e.g. Bernoulli traits) in the absence of repeated measurements within a given environmental context C (e.g. a given year).

### 2.2 Validation on simulated datasets

We validated the CRN model on two different types of trade‐offs. First, we used the hybrid CRN model to study an intergenerational trade‐off between fecundity and quality. The hybrid model is well suited because fecundity (i.e. clutch/litter size) has a single measurement per mother per year, while offspring quality (i.e. offspring mass) has repeated measurements per mother per year (one measurement for each offspring produced). Second, we used the non‐repeated measures CRN model to study an intraindividual trade‐off between fecundity (clutch/litter size) and parental growth (the change of mass from a year to the next). The non‐repeated measures CRN model is well suited as both traits are expressed only a single time per year (one fecundity and one parental growth measure per individual per year). Note that trade‐offs are described as intergenerational or intraindividual depending on which traits are studied (as explained in Stearns, 1989), and both type of trade‐off can be decomposed into among‐ and within‐individual covariation. We simulate data for these two trade‐offs using the individual‐based simulation described in Bliard, Paniw, et al. (2024), whereby the among‐individual correlation between life history traits can be made dependent on the environmental context. The code to generate data from the individual‐based simulation can be found on Zenodo (Bliard, Martin, et al., 2024). This model validation is only intended to show that context‐dependent among‐individual correlations (i.e. context dependent trade‐offs) can be successfully recovered. For a more extensive simulation‐based calibration of CRN models over a broad range of parameter values, see Martin (2023).

#### 2.2.1 Intergenerational trade‐off (offspring quantity‐quality)

We first focused on an intergenerational trade‐off between offspring quantity and quality (hybrid CRN model). This quantity‐quality trade‐off has been the focus of numerous studies since Lack's pioneering work on bird clutch sizes (Einum & Fleming, 2000; Fischer et al., 2011; Gillespie et al., 2008; Lack, 1947; Williams, 1966). We simulate 30 years of individual‐based data in which 25 new individuals enter the population each year, reproduce with an average clutch/litter size of 2.5, and then have a probability to survive to next year of 0.6. This yielded a final simulated dataset of 750 individuals, totaling 1578 reproductive events and 4783 offspring. An observation‐level correlation was included between offspring mass and clutch size, and this correlation was made dependent on a single climatic predictor. The same climatic predictor was also included to influence both clutch size and offspring mass.

#### 2.2.2 Intraindividual trade‐off (fecundity‐growth)

We then simulated data for an intraindividual trade‐off between fecundity and growth (non‐repeated measures CRN model). This simulated dataset is also made of 30 years and 750 individuals, for a total of 1974 reproductive events, with a variable observation‐level correlation between individual growth and fecundity, which is itself dependent on a single climatic predictor.

### 2.3 Study systems and application on empirical datasets

#### 2.3.1 Marmots

We applied the hybrid CRN model (one trait with repeated individual measurements within a year and one trait without) on data from a yellow‐bellied marmot population monitored at the Rocky Mountain Biological Laboratory in Gothic, Colorado (38°57′ N, 106°59′ W) during the summer season each year, whereby extensive individual‐based data is collected (Armitage, 2014; Blumstein, 2013). Data were collected under the UCLA Institutional Animal Care and Use protocol (2001‐191‐01, renewed annually) and with permission from the Colorado Parks and Wildlife (TR917, renewed annually). The research was in compliance with ethical guidelines and current laws of the USA and the State of Colorado. In Alpine marmots, *Marmota marmota*, an offspring quality‐quantity trade‐off has been found (Berger et al., 2015), while it remained mostly elusive in yellow‐bellied marmots, being only found for older mothers (Kroeger et al., 2020), whereby within‐cohort selection has likely reduced the amount of among‐individual variance in resource acquisition, thus making the trade‐off visible (Kendall et al., 2011; van Noordwijk & de Jong, 1986). Therefore, we searched for an intergenerational trade‐off between mothers' fecundity and offspring estimated mass (offspring quality‐quantity trade‐off). We used repeated measurements of offspring mass for each mother (one mass estimate for each offspring in a given litter). The offspring weaning mass was imputed based on the date of emergence for each litter and mass measurements from captures later in the season, following the method of Ozgul et al. (2010). We considered two measures quantifying environmental conditions for a given year. First, the total amount of snow during the preceding winter, with years of little overwinter snow considered harsher for marmots as it offers limited thermal insulation during the hibernation (Barash, 1973; Cordes et al., 2020; Wells et al., 2022). Second, the average daily maximum temperature during the month of June, with warmer summer temperatures considered unfavourable conditions for marmots as they are prone to overheating, hence limiting the time that can be allocated to foraging (Cordes et al., 2020; Krajick, 2004; Melcher et al., 1990). Note that we used temperature in June and not July as commonly used in this system (Cordes et al., 2020), because this is more likely to represent the conditions experienced for most offspring before emergence and weaning, since most offspring emerge in July. We expected trade‐offs to be more strongly expressed among individuals in years with little overwinter snow or high summer temperature. In total, we used 2540 offspring mass from 597 reproductive events, from 279 females across 42 years.

We modelled offspring mass using a normal distribution (Equation 6.1), and we included as covariates (i.e. in **X**_**1**_) the total amount of snow during the winter, June average maximum temperature, age of the mother and its quadratic effect, and mother's estimated mass in early June as a proxy of mother's quality. A year random effect δ_1_ was also included,

(6.1) $$\text{offpsring mass}=X_{1}\beta_{1}+\delta_{1}+{\mathrm{W\alpha}}_{1\left(Y\right)}+\epsilon_{1\left(Y\right)}$$

With $a_{1\left(Y\right)}$ being a year‐specific mother random effect and $\epsilon_{1\left(Y\right)}$ the within‐litter variance.

We modelled the second trait, fecundity (i.e. litter size), using a Poisson distribution (Equation 6.2), as a function of the same covariates (**X**_**2**_), except June average maximum temperature, since it cannot affect fecundity as pregnancies mostly occur before this period. A year random effect δ_2_ was also included,

(6.2) $$\log\left(\text{litter size}\right)=X_{2}\beta_{2}+\delta_{2}+o_{2\left(Y\right)}$$

For the observation‐level correlation (Equation 6.3), the two environmental variables (winter snow and June temperature) were added as covariates (**X**_**3**_),

(6.3) $$\begin{array}{c} \left[\alpha_{1\left(Y\right)},o_{2\left(Y\right)}\right]\sim N\left(0,P_{\left(Y\right)}\right),\\ P_{\left(Y\right)}=\left[\begin{array}{cc} \sigma_{\alpha_{1}\left(Y\right)}^{2} & r_{\left(X\right)}\sigma_{\alpha_{1}\left(Y\right)}\sigma_{o_{2}\left(Y\right)}\\ r_{\left(Y\right)}\sigma_{\alpha_{1}\left(Y\right)}\sigma_{o_{2}\left(Y\right)} & \sigma_{o_{2}\left(Y\right)}^{2} \end{array}\right],\\ \text{atanh}\left(r_{\left(Y\right)}\right)=X_{3}\beta_{3}. \end{array}$$

We performed posterior predictive checks, showing a good concordance between the litter size data, and data generated under the model (see Figure S3). However, the model slightly underestimates the variance in offspring mass. Overall, posterior predictive checks highlight that the use of a Normal distribution to model offspring mass and a Poisson distribution with an observation random effect to model litter size, were appropriate in this system.

#### 2.3.2 Soay sheep

We applied the non‐repeated measures CRN model on Soay sheep data, as we have no repeated individual measurement within a given year available for neither of the traits studied. We used data from an unmanaged population of feral sheep in the Village Bay area of the island of Hirta (57°48′ N, 8°37′ W), which has been monitored since 1985 (Clutton‐Brock & Pemberton, 2004). All fieldwork was conducted in accordance with the Animals (Scientific Procedures) Act 1986 and with permission from the University of Edinburgh Animal Welfare Ethical Review Body. In Soay sheep, survival costs of reproduction were found for breeding ewes, particularly in populations at high densities or following stormy winters (Tavecchia et al., 2005). Therefore, we searched for an intraindividual trade‐off between ewes' fecundity defined as the number of lambs born in Spring (ranging from 0 to 2) and their log mass in the following summer, with both traits conditional on ewes surviving the winter. We considered two environmental variables to characterise the ecological harshness faced by the sheep in a given year: population density and NAO (North Atlantic Oscillation) in the winter preceding parturition, with high NAO values corresponding to wet and stormy winters (Coulson et al., 2001; Regan et al., 2022). In total, we used data from 2497 reproductive events across 37 years, for 861 ewes with known mass in the summer preceding the reproductive event, as well as known mass in the following summer. We expected trade‐offs to be more strongly expressed in years of high population density or high NAO.

As ewes' fecundity in a given year is restricted to [0,2], we could not use a Poisson regression. This is due to the count data being underdispersed relative to a Poisson distribution. We therefore modelled the ewe's fecundity using an ordinal regression (also called cumulative logistic regression; Equation 7.1), and we included as covariates (**X**_**1**_) the individual's log mass preceding the reproductive event as a proxy of quality, age and its quadratic effect, and population density,

(7.1) $$\text{logit}\left(\mathrm{Pr}\left(N_{\text{offspring}}\leq i\right)\right)=\theta_{i}-\left(X_{1}\beta_{1}+\delta_{1}+o_{1\left(Y\right)}\right)$$

where the cumulative probability of having at most *i* offspring is given as a function of the threshold $\theta_{i}$ and the matrix of covariates **X**_**1**_, as well as a year random effect δ_1_ and a year specific observation random effect $o_{1\left(Y\right)}$. We modelled the ewe's log mass in the following summer using a normal distribution (Equation 7.2), and included in **X**_**2**_ the same covariates as in **X**_**1**_, as well as NAO in the winter preceding parturition. A year random effect δ_2_ was also included.

(7.2) $$\text{mass}=X_{2}\beta_{2}+\delta_{2}+o_{2\left(Y\right)}$$

For the observation‐level correlation (Equation 7.3), the two ecological variables (winter NAO and density) were added as covariates (**X**_**3**_),

(7.3) $$\begin{array}{c} \left[o_{1\left(Y\right)},o_{2\left(Y\right)}\right]\sim N\left(0,P_{\left(Y\right)}\right)\\ P_{\left(Y\right)}=\left[\begin{array}{cc} \sigma_{o_{1}\left(Y\right)}^{2} & r_{\left(Y\right)}\sigma_{o_{1}\left(Y\right)}\sigma_{o_{2}\left(Y\right)}\\ r_{\left(Y\right)}\sigma_{o_{1}\left(Y\right)}\sigma_{o_{2}\left(Y\right)} & \sigma_{o_{2}\left(Y\right)}^{2} \end{array}\right]\\ \text{atanh}\left(r_{\left(Y\right)}\right)=X_{3}\beta_{3} \end{array}$$

The posterior predictive checks we performed highlighted a good fit between the data and data generated under the model. This confirms that using a normal distribution to model ewe's mass, and using a cumulative logistic regression to model ewe's number of offspring, were appropriate (see Figure S4).

### 2.4 Model implementation

We implemented all multivariate models described above in a Bayesian framework using the Stan statistical language (Carpenter et al., 2017), through the software R (R Core Team, 2021) using the R package *CmdStanR* (Gabry & Češnovar, 2020). Stan was preferred for model implementation because of its flexibility. Common regularising priors were used for all model parameters: normal distributions of mean 0 and standard deviation of 1 for intercepts and slopes coefficients, and exponential distributions of rate 2 for variance parameters. Each model ran on 3 chains, with a burn‐in period of 1000 iterations, sampling for 3000 iterations, keeping all the sampled iterations (Link & Eaton, 2012). Convergence of parameter estimates was assessed visually and using the Gelman‐Rubin diagnostic (Gelman & Rubin, 1992). We report the full posterior distributions, alongside their mean, 50%, and 89% credible intervals (McElreath, 2020). The Stan code to implement all the CRN models presented in this study is available on GitHub (https://github.com/lbiard/detecting_tradeoffs_crn_models) and is permanently archived on Zenodo (https://doi.org/10.5281/zenodo.12800618).

## 3 RESULTS

**FIGURE 2:**
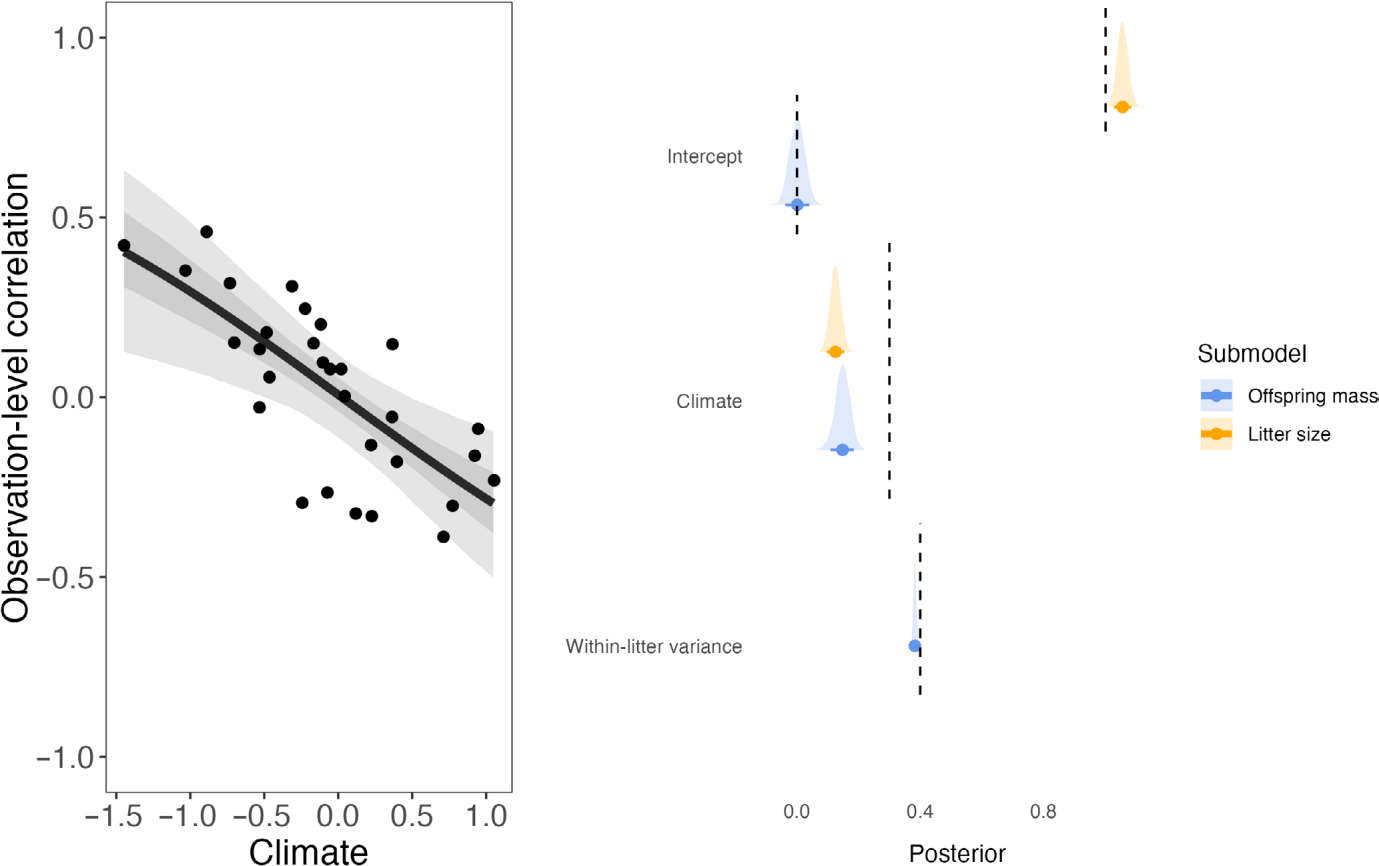
Left panel: Estimated versus simulated observation‐level correlation between litter size and offspring mass as a function of climate, after accounting for the effect of climate on both traits. The regression line indicates the mean effect of climate on the correlation, while the shaded areas depict the 50% and 89% credible intervals predicted by the model. Each black dot represents the simulated observation‐level correlation between both traits in a given year depending on climate. Right panel: Estimated versus simulated intercepts and slopes for the offspring mass and litter size sub‐models. Dashed lines represent the value used to simulate the data, while the distributions and intervals represent the posterior distributions estimated by the model, alongside the median, 50% and 89% credible intervals. Litter size estimates are presented on the log scale.

**FIGURE 3:**
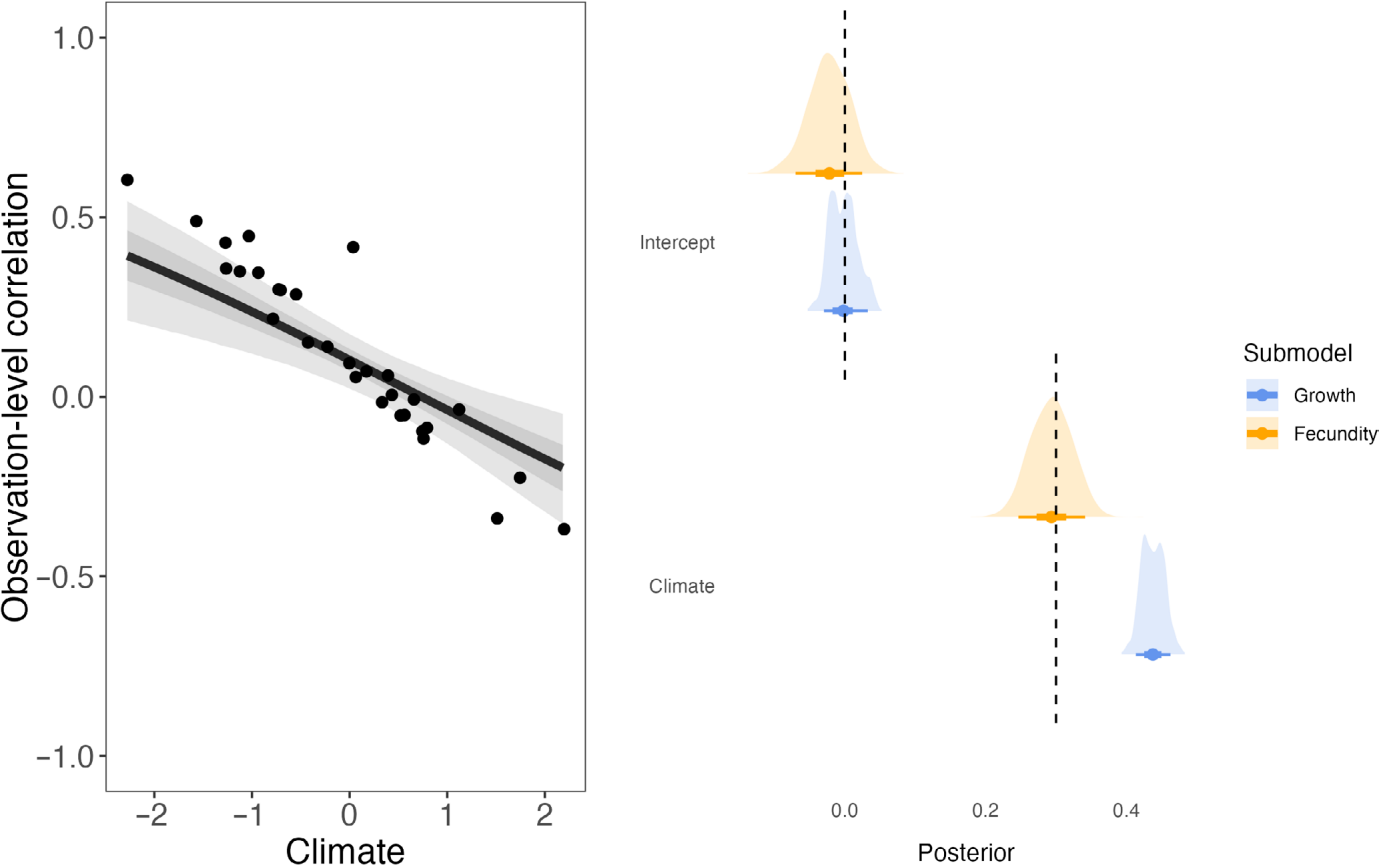
Left panel: Estimated versus simulated observation‐level correlation between fecundity and growth as a function of climate, after accounting for the effect of climate on both traits. The regression line indicates the mean effect of climate on the correlation, while the shaded areas depict the 50% and 89% credible intervals predicted by the model. Each black dot represents the simulated observation‐level correlation between both traits in a given year depending on climate. Right panel: Estimated versus simulated intercepts and slopes for the growth and fecundity sub‐models. Dashed lines represent the value used to simulate the data, while the distributions and intervals represent the posterior distributions estimated by the model, alongside the median, 50%, 89% credible intervals. Fecundity estimates are presented on the log scale.

The model validation performed on simulated datasets showed that parameters were correctly recovered for both intergenerational trade‐offs (Figure 2) and intraindividual trade‐offs (Figure 3). While these simulation examples do not quantify bias of estimations (more details from a simulation‐based calibration of CRN models are available in Martin, 2023), they still confirm that the model presented in the methods is able to detect context‐dependence in the expression of trade‐offs.

**FIGURE 4:**
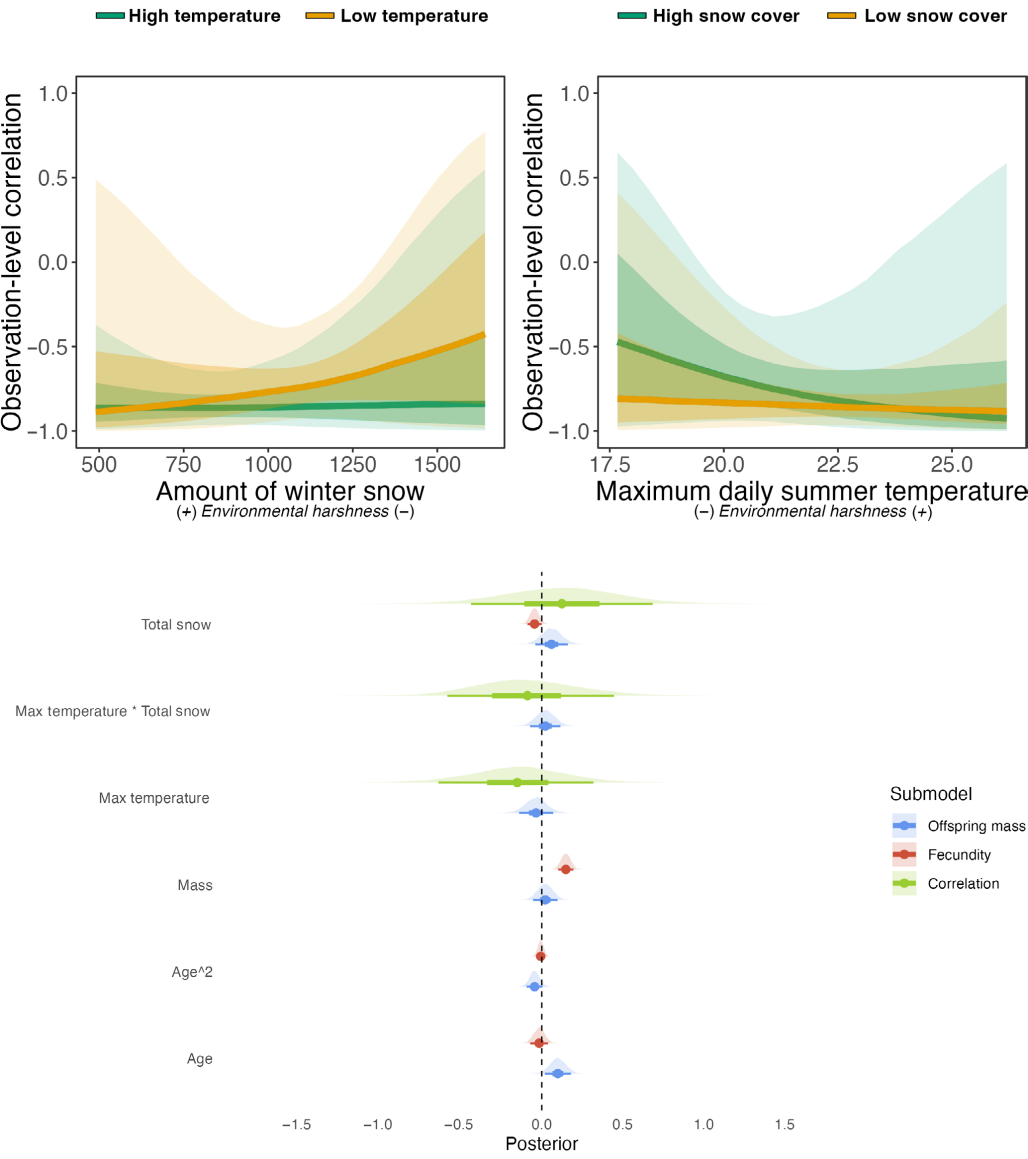
Observation‐level correlation between litter size and offspring mass in marmots as a function of the total amount of snow in the preceding winter at high and low temperature (top left panel) and the maximum daily June temperature of the year at high and low snow cover (top right panel). Estimated effects of standardised predictors (bottom panel) on offspring mass, fecundity, and the observation‐level correlation between both traits in marmots. The regression line indicates the median estimated effect, while the shaded areas depict the 50% and 89% credible intervals predicted by the model.

The model applied to yellow‐bellied marmot data shows trends towards trade‐offs being more strongly expressed in years with harsh environmental conditions, albeit with high uncertainty in the estimates (Figure 4). We found a positive mean effect of the amount of overwinter snow on the correlation (Figure 4), meaning that the trade‐off between fecundity and offspring quality was more strongly expressed after winters with little snow. We also found a negative mean effect of the average maximum June temperature on the correlation (Figure 4), where females with more offspring were more likely to have lighter offspring during warmer summers. Estimated effects of covariates on either fecundity or offspring mass can be found in Figure 4, as well as in Figure S5.

**FIGURE 5:**
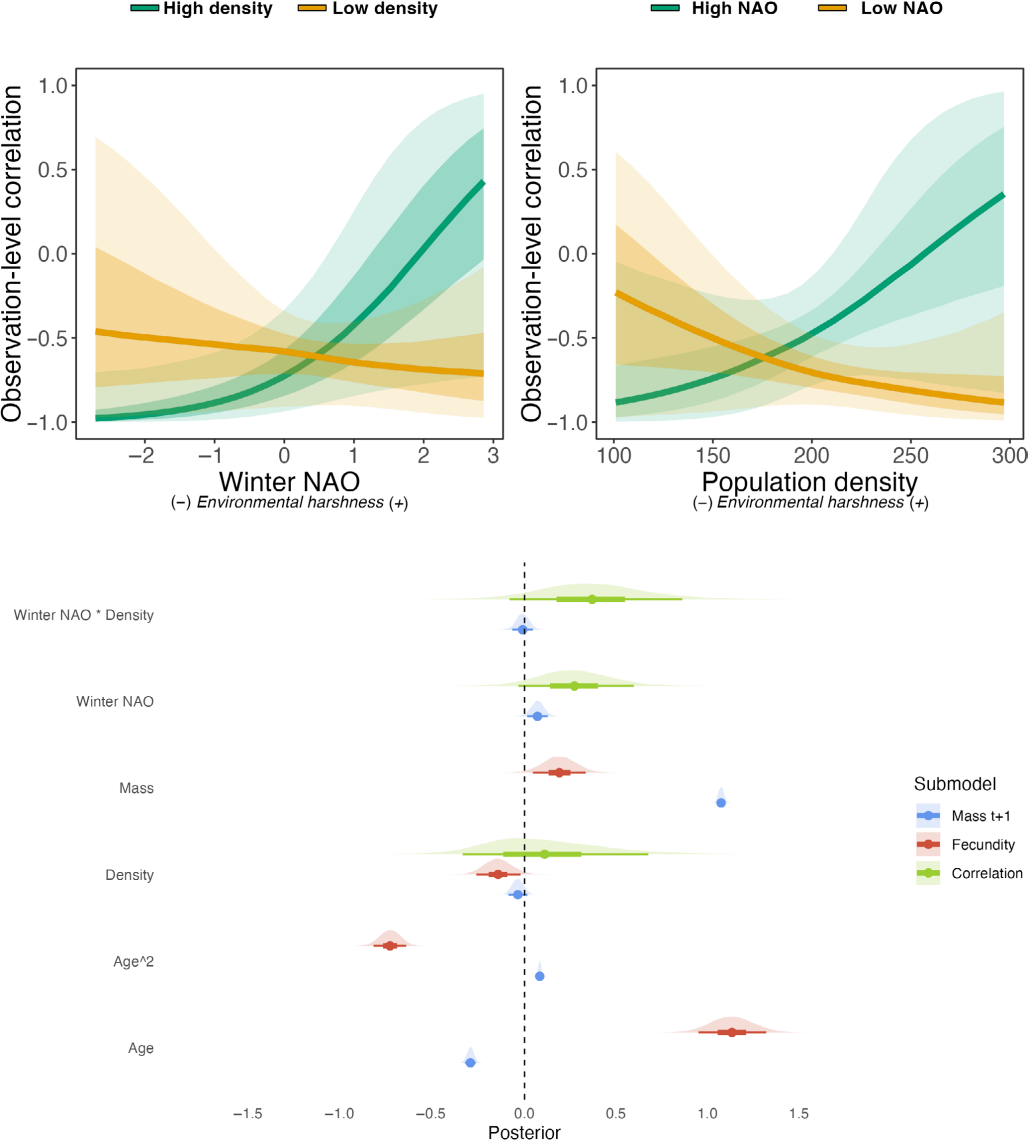
Observation‐level correlation between fecundity and mothers' mass in the following year in Soay sheep as a function of the winter NAO at high and low density (top left panel), and as a function of the population density at high and low winter NAO values (top right panel). Estimated effects (bottom panel) of standardised predictors on mother's mass in the following year, fecundity, and the observation‐level correlation between both traits in Soay sheep. The figure displays the posterior distributions estimated by the model, alongside the median, 50% and 89% credible intervals.

Estimated effects of covariates on the correlation also had high uncertainty in the Soay sheep dataset (Figure 5). Overall, we found that the correlation tended to be negative across most environments, which means that ewe's growth was lower for the ones that weaned offspring (Figure 5). Contrary to our expectations, while we hypothesised that the trade‐offs should be more strongly expressed in wet and stormy winters (high NAO index), we found a positive effect of winter NAO on the correlation between fecundity and growth (Figure 5). We also found a positive effect of population density on the expression of the trade‐off (Figure 5). Estimated effects of covariates on either fecundity or ewe's mass can be found in Figure 5, as well as in Figure S6.

## 4 DISCUSSION

Our proof‐of‐concept study demonstrates that hierarchical multivariate CRN models (Martin, 2023) can be used successfully to detect and estimate context‐dependent changes in trade‐off expression, though estimation uncertainty can be large. In agreement with theoretical predictions and despite large uncertainty, we found that reproductive trade‐offs in yellow‐bellied marmots tend to be more strongly expressed under unfavourable climatic conditions. In Soay sheep, we found some context‐dependence in the expression of the trade‐off, but effect directions were opposite to our initial prediction. This hierarchical model has the potential to be used on many long‐term individual‐based datasets and could help improve our understanding of trade‐off expression and life history theory.

Although the initial motivation to use this method partly rested on the observed difficulty of finding trade‐offs in empirical datasets, we found that in both sheep and marmots, the trade‐offs tend to be expressed across most environments, with mean phenotypic correlations being negative overall. Thus, ironically, in these two empirical datasets, trade‐offs might have been detected using simpler multivariate methods without the need for context dependence. However, this should not come as a surprise for Soay sheep, as this negative correlation between growth and fecundity was already found on a smaller dataset (Fung et al., 2022). Nonetheless, the results still highlight that context‐dependence has the potential to hinder our ability to detect trade‐offs in some cases. For instance, when marmots experience favourable environmental conditions, the average correlation is closer to null with credibility intervals nearing or overlapping zero (Figure 4), while this intergenerational trade‐off is found to be more strongly expressed during harsh years. In Soay sheep, context dependence appears to be marked for the expression of the trade‐off, but opposite to our predictions. Indeed, we found a positive correlation between growth and fecundity only under the harshest environmental conditions (high population density and high winter NAO, Figure 5). Since ewes' mass is measured in the following summer and not directly after parturition, harsh winter conditions are expected to increase overwinter mortality (Milner et al., 1999), lowering spring population density and reducing competition. This could potentially help surviving ewes to recover their body condition between spring and summer, which is the period of greatest grass growth, hence potentially explaining our counter‐intuitive results. We can also speculate that the result could have arisen from two potential pitfalls due to idiosyncrasies of the Soay sheep data. First, among‐individual variation in fecundity is limited in sheep, ranging from no offspring to twins, potentially making it more complicated for the model to estimate variances accurately (Fay, Authier, et al., 2022; Kain et al., 2015). Second, both ewes' growth and fecundity are conditional on survival in the data, hence individuals who suffered most from the cost of reproduction and did not survive are not present in the analysis, potentially biasing the results (Hadfield, 2008). Finally, while we expected more negative phenotypic correlations under harsh conditions, where among‐individual variance in resource allocation is greater than among‐individual variance in acquisition, it is theoretically possible that in a population facing adverse conditions, a few robust individuals monopolise most resources, thus increasing the among‐individual variance in resource acquisition (Chambert et al., 2013), hence leading to positive estimates of phenotypic correlations.

Despite the potential of this modelling approach to study context‐dependent trade‐offs, a few methodological limitations are to be considered. A recent study conducted by Fay, Authier, et al. (2022) highlighted that multivariate models with correlated random effects for Bernoulli traits performed rather poorly, resulting in a potentially large bias and imprecise estimates of variances and covariances. This is in part because Bernoulli traits contain less information than continuous variables, making estimations of variances complicated (Fay, Authier, et al., 2022), but also because the data available to estimate individual heterogeneity is usually scarce (Browne et al., 2007). The model we present suffers from this limitation, and even more so when there is only a single individual observation per individual per sampling occasion (e.g. parental survival), and when the trait is not repeatable (death can only occur once). This issue renders the model, as well as any other multilevel model, unable to meaningfully estimate distinct mean and variance parameters for Bernoulli traits, due to the fact that the mean *p* of a Bernoulli variable determines its variability *p*(1‐*p*) without scope for overdispersion. Therefore, environmental effects on the mean of Bernoulli measures will necessarily change their variances (Skrondal & Rabe‐Hesketh, 2007). However, when repeated Bernoulli observations or a binomial measure are available within each sampling occasion (e.g. survival of each offspring within a litter), the CRN model can then be used to partition distinct environmental effects on trait means and (co)variances. As we have shown in the present study, despite this limitation, the CRN remains applicable to single measures of continuous traits and count measures (e.g. growth, fecundity, phenology, behavioural traits), as well as proportions and various other kinds of non‐Gaussian measures. Another limitation of the proposed method is that sample sizes needed are likely to be large, with enough individuals in each environmental context, and importantly enough sampling occasions across which to estimate the context dependence of trade‐off expression. Nonetheless, many long‐term individual‐based studies should have enough data to fulfil these requirements (de Villemereuil et al., 2020).

Despite the abovementioned caveats and limitations of the methodology in the absence of repeated measurements, this new model is a development that could be useful for many datasets. Thanks to its implementation in a Bayesian framework using Stan (Carpenter et al., 2017), it offers great flexibility and can be easily repurposed and modified to fit the idiosyncrasies of a given dataset or species life history. It is also straightforward to extend the model by adding a pedigree for quantitative genetic analysis (see Martin, 2023), even though phenotypic correlations should be good approximations of genetic correlations in most cases (Cheverud, 1988; Dochtermann, 2011; Roff, 1995). While we presented a bivariate model, this model is not necessarily limited to two traits, and more continuous traits and their covariances could also be analysed simultaneously. We also restricted our proof‐of‐concept study to the reaction norm of the correlation between traits, but researchers interested in the canalisation of traits variances as a response to the environmental context could also benefit from this modelling approach (Péron et al., 2016).

Life history trade‐offs have long been sought after, but difficult to detect in observational data due to individual heterogeneity (Metcalf, 2016; Reznick et al., 2000; van Noordwijk & de Jong, 1986). Previous studies have also highlighted that life history trade‐offs could be expressed only under unfavourable ecological conditions (Cohen et al., 2020; Stearns, 1989). Yet, despite our knowledge of the issues hindering trade‐off detection, we still lacked a statistical framework that permits the detection of context‐dependence in trade‐off expression. Our proof‐of‐concept study shows that this context dependence can be detected. This method has the potential to be applied by demographers and evolutionary ecologists having long‐term individual‐based datasets at hands, with many study systems having the required data (Culina et al., 2021; de Villemereuil et al., 2020). Altogether, this method has the potential to help us improve our understanding of life history theory, and in part resolve van Noordwijk and de Jong (1986) conundrum of trade‐off detection, by accounting for the context‐dependence of their expression.

## AUTHOR CONTRIBUTIONS

Louis Bliard, Jordan S. Martin, Arpat Ozgul, Maria Paniw and Dylan Z. Childs conceived the study. Jordan S. Martin designed the initial modelling framework and Louis Bliard analysed the data. Daniel T. Blumstein, Julien G.A. Martin, Josephine M. Pemberton, Daniel H. Nussey collected and curated the data. Louis Bliard and Jordan S. Martin wrote the first draft with input from Arpat Ozgul, Maria Paniw and Dylan Z. Childs. All authors contributed to the editing of the manuscript.

## CONFLICT OF INTEREST STATEMENT

The authors declare no conflicts of interest.

## Supporting information


**Section S1.** Fixed individual heterogeneity.
**Section S2.** Posterior predictive checks.
**Section S3.** Associations between the covariates and traits studied.

## Data Availability

The data, as well as the R and Stan code necessary to reproduce the results are available on GitHub https://github.com/lbiard/detecting_tradeoffs_crn_models and are archived on Zenodo https://doi.org/10.5281/zenodo.12800618 (Bliard, Martin, et al., 2024).

## References

[jane14173-bib-0001] Agrawal, A. A. (2020). A scale‐dependent framework for trade‐offs, syndromes, and specialization in organismal biology. Ecology, 101(2), e02924. 10.1002/ecy.2924 31660584

[jane14173-bib-0002] Armitage, K. B. (2014). Marmot biology: Sociality, individual fitness, and population dynamics. Cambridge University Press. 10.1017/CBO9781107284272 22017671

[jane14173-bib-0003] Barash, D. P. (1973). The social biology of the Olympic marmot. Animal Behaviour Monographs, 6, 171–245. 10.1016/0003-3472(73)90002-X

[jane14173-bib-0004] Bell, A. M. , Hankison, S. J. , & Laskowski, K. L. (2009). The repeatability of behaviour: A meta‐analysis. Animal Behaviour, 77(4), 771–783. 10.1016/j.anbehav.2008.12.022 24707058 PMC3972767

[jane14173-bib-0005] Berger, V. , Lemaître, J.‐F. , Gaillard, J.‐M. , & Cohas, A. (2015). How do animals optimize the size–number trade‐off when aging? Insights from reproductive senescence patterns in marmots. Ecology, 96(1), 46–53. 10.1890/14-0774.1 26236889

[jane14173-bib-0006] Bielby, J. , Mace, G. M. , Bininda‐Emonds, O. R. P. , Cardillo, M. , Gittleman, J. L. , Jones, K. E. , Orme, C. D. L. , & Purvis, A. (2007). The fast‐slow continuum in mammalian life history: An empirical reevaluation. The American Naturalist, 169(6), 748–757. 10.1086/516847 17479461

[jane14173-bib-0007] Bliard, L. , Martin, J. S. , Paniw, M. , Blumstein, D. T. , Martin, J. G. A. , Pemberton, J. M. , Nussey, D. H. , Childs, D. Z. , & Ozgul, A. (2024). Data and code for “detecting context‐dependence in the expression of life history tradeoffs” [data set]. *Zenodo*. 10.5281/zenodo.12800618 PMC11880661 39221784

[jane14173-bib-0008] Bliard, L. , Paniw, M. , Childs, D. Z. , & Ozgul, A. (2024). Population dynamic consequences of context‐dependent tradeoffs across life histories. The American Naturalist, 203(6), 681–694. 10.1086/730111 38781530

[jane14173-bib-0009] Blumstein, D. T. (2013). Yellow‐bellied marmots: Insights from an emergent view of sociality. Philosophical Transactions of the Royal Society, B: Biological Sciences, 368(1618), 20120349. 10.1098/rstb.2012.0349 PMC3638452 23569297

[jane14173-bib-0010] Browne, W. J. , McCleery, R. H. , Sheldon, B. C. , & Pettifor, R. A. (2007). Using cross‐classified multivariate mixed response models with application to life history traits in great tits (*Parus major*). Statistical Modelling, 7(3), 217–238. 10.1177/1471082X0700700301

[jane14173-bib-0011] Cam, E. , Link, W. A. , Cooch, E. G. , Monnat, J. , & Danchin, E. (2002). Individual covariation in life‐history traits: Seeing the trees despite the forest. The American Naturalist, 159(1), 96–105. 10.1086/324126 18707403

[jane14173-bib-0092] Cam, E. , Gimenez, O. , Alpizar‐Jara, R. , Aubry, L. M. , Authier, M. , Cooch, E. G. , Koons, D. N. , Link, W. A. , Monnat, J. , Nichols, J. D. , Rotella, J. J. , Royle, J. A. , & Pradel, R. (2013). Looking for a needle in a haystack: Inference about individual fitness components in a heterogeneous population. Oikos, 122(5), 739–753. 10.1111/j.1600-0706.2012.20532.x

[jane14173-bib-0012] Carpenter, B. , Gelman, A. , Hoffman, M. D. , Lee, D. , Goodrich, B. , Betancourt, M. , Brubaker, M. , Guo, J. , Li, P. , & Riddell, A. (2017). Stan: A probabilistic programming language. Journal of Statistical Software, 76, 1–32. 10.18637/jss.v076.i01 36568334 PMC9788645

[jane14173-bib-0013] Cauchoix, M. , Chow, P. K. Y. , van Horik, J. O. , Atance, C. M. , Barbeau, E. J. , Barragan‐Jason, G. , Bize, P. , Boussard, A. , Buechel, S. D. , Cabirol, A. , Cauchard, L. , Claidière, N. , Dalesman, S. , Devaud, J. M. , Didic, M. , Doligez, B. , Fagot, J. , Fichtel, C. , Henke‐von der Malsburg, J. , … Morand‐Ferron, J. (2018). The repeatability of cognitive performance: A meta‐analysis. Philosophical Transactions of the Royal Society, B: Biological Sciences, 373(1756), 20170281. 10.1098/rstb.2017.0281 PMC6107569 30104426

[jane14173-bib-0014] Chambert, T. , Rotella, J. J. , Higgs, M. D. , & Garrott, R. A. (2013). Individual heterogeneity in reproductive rates and cost of reproduction in a long‐lived vertebrate. Ecology and Evolution, 3(7), 2047–2060. 10.1002/ece3.615 23919151 PMC3728946

[jane14173-bib-0015] Chang, C. , Moiron, M. , Sánchez‐Tójar, A. , Niemelä, P. T. , & Laskowski, K. L. (2023). What is the meta‐analytic evidence for life‐history trade‐offs at the genetic level? Ecology Letters, 27, ele.14354. 10.1111/ele.14354 38115163

[jane14173-bib-0016] Cheverud, J. M. (1988). A comparison of genetic and phenotypic correlations. Evolution, 42(5), 958–968. 10.1111/j.1558-5646.1988.tb02514.x 28581166

[jane14173-bib-0017] Clutton‐Brock, T. H. , & Pemberton, J. M. (2004). Soay sheep dynamics and selection in an Island population. Cambridge University Press.

[jane14173-bib-0018] Cohen, A. A. , Coste, C. F. D. , Li, X.‐Y. , Bourg, S. , & Pavard, S. (2020). Are trade‐offs really the key drivers of ageing and life span? Functional Ecology, 34(1), 153–166. 10.1111/1365-2435.13444

[jane14173-bib-0019] Compagnoni, A. , Bibian, A. J. , Ochocki, B. M. , Rogers, H. S. , Schultz, E. L. , Sneck, M. E. , Elderd, B. D. , Iler, A. M. , Inouye, D. W. , Jacquemyn, H. , & Miller, T. E. X. (2016). The effect of demographic correlations on the stochastic population dynamics of perennial plants. Ecological Monographs, 86(4), 480–494. 10.1002/ecm.1228

[jane14173-bib-0020] Cordes, L. S. , Blumstein, D. T. , Armitage, K. B. , CaraDonna, P. J. , Childs, D. Z. , Gerber, B. D. , Martin, J. G. A. , Oli, M. K. , & Ozgul, A. (2020). Contrasting effects of climate change on seasonal survival of a hibernating mammal. Proceedings of the National Academy of Sciences of the United States of America, 117(30), 18119–18126. 10.1073/pnas.1918584117 32631981 PMC7395557

[jane14173-bib-0021] Coulson, T. , Catchpole, E. A. , Albon, S. D. , Morgan, B. J. T. , Pemberton, J. M. , Clutton‐Brock, T. H. , Crawley, M. J. , & Grenfell, B. T. (2001). Age, sex, density, winter weather, and population crashes in Soay Sheep. Science, 292(5521), 1528–1531. 10.1126/science.292.5521.1528 11375487

[jane14173-bib-0022] Cressler, C. E. , Bengtson, S. , & Nelson, W. A. (2017). Unexpected nongenetic individual heterogeneity and trait covariance in daphnia and its consequences for ecological and evolutionary dynamics. The American Naturalist, 190(1), E13–E27. 10.1086/691779 28617635

[jane14173-bib-0023] Culina, A. , Adriaensen, F. , Bailey, L. D. , Burgess, M. D. , Charmantier, A. , Cole, E. F. , Eeva, T. , Matthysen, E. , Nater, C. R. , Sheldon, B. C. , Sæther, B.‐E. , Vriend, S. J. G. , Zajkova, Z. , Adamík, P. , Aplin, L. M. , Angulo, E. , Artemyev, A. , Barba, E. , Barišić, S. , … Visser, M. E. (2021). Connecting the data landscape of long‐term ecological studies: The SPI‐birds data hub. Journal of Animal Ecology, 90(9), 2147–2160. 10.1111/1365-2656.13388 33205462 PMC8518542

[jane14173-bib-0024] de Jong, G. (1993). Covariances between traits deriving from successive allocations of a resource. Functional Ecology, 7(1), 75–83. 10.2307/2389869

[jane14173-bib-0025] de Jong, G. , & van Noordwijk, A. J. (1992). Acquisition and allocation of resources: Genetic (CO) variances, selection, and life histories. The American Naturalist, 139(4), 749–770.

[jane14173-bib-0026] de Villemereuil, P. , Charmantier, A. , Arlt, D. , Bize, P. , Brekke, P. , Brouwer, L. , Cockburn, A. , Côté, S. D. , Dobson, F. S. , Evans, S. R. , Festa‐Bianchet, M. , Gamelon, M. , Hamel, S. , Hegelbach, J. , Jerstad, K. , Kempenaers, B. , Kruuk, L. E. B. , Kumpula, J. , Kvalnes, T. , … Chevin, L.‐M. (2020). Fluctuating optimum and temporally variable selection on breeding date in birds and mammals. Proceedings of the National Academy of Sciences of the United States of America, 117(50), 31969–31978. 10.1073/pnas.2009003117 33257553 PMC7116484

[jane14173-bib-0027] Descamps, S. , Gaillard, J.‐M. , Hamel, S. , & Yoccoz, N. G. (2016). When relative allocation depends on total resource acquisition: Implication for the analysis of trade‐offs. Journal of Evolutionary Biology, 29(9), 1860–1866. 10.1111/jeb.12901 27200492

[jane14173-bib-0028] Dingemanse, N. J. , Araya‐Ajoy, Y. G. , & Westneat, D. F. (2021). Most published selection gradients are underestimated: Why this is and how to fix it. Evolution, 75(4), 806–818. 10.1111/evo.14198 33621355

[jane14173-bib-0029] Dingemanse, N. J. , & Dochtermann, N. A. (2013). Quantifying individual variation in behaviour: Mixed‐effect modelling approaches. Journal of Animal Ecology, 82(1), 39–54. 10.1111/1365-2656.12013 23171297

[jane14173-bib-0030] Dochtermann, N. A. (2011). Testing Cheverud's conjecture for behavioral correlations and behavioral syndromes. Evolution, 65(6), 1814–1820. 10.1111/j.1558-5646.2011.01264.x 21644966

[jane14173-bib-0031] Einum, S. , & Fleming, I. A. (2000). Highly fecund mothers sacrifice offspring survival to maximize fitness. Nature, 405(6786), 565–567. 10.1038/35014600 10850714

[jane14173-bib-0032] Fay, R. , Authier, M. , Hamel, S. , Jenouvrier, S. , van de Pol, M. , Cam, E. , Gaillard, J.‐M. , Yoccoz, N. G. , Acker, P. , Allen, A. , Aubry, L. M. , Bonenfant, C. , Caswell, H. , Coste, C. F. D. , Larue, B. , Le Coeur, C. , Gamelon, M. , Macdonald, K. R. , Moiron, M. , … Sæther, B.‐E. (2022). Quantifying fixed individual heterogeneity in demographic parameters: Performance of correlated random effects for Bernoulli variables. Methods in Ecology and Evolution, 13(1), 91–104. 10.1111/2041-210X.13728

[jane14173-bib-0033] Fay, R. , Hamel, S. , van de Pol, M. , Gaillard, J.‐M. , Yoccoz, N. G. , Acker, P. , Authier, M. , Larue, B. , Le Coeur, C. , Macdonald, K. R. , Nicol‐Harper, A. , Barbraud, C. , Bonenfant, C. , Van Vuren, D. H. , Cam, E. , Delord, K. , Gamelon, M. , Moiron, M. , Pelletier, F. , … Sæther, B.‐E. (2022). Temporal correlations among demographic parameters are ubiquitous but highly variable across species. Ecology Letters, 25(7), 1640–1654. 10.1111/ele.14026 35610546 PMC9323452

[jane14173-bib-0034] Fay, R. , Michler, S. , Laesser, J. , Jeanmonod, J. , & Schaub, M. (2020). Can temporal covariation and autocorrelation in demographic rates affect population dynamics in a raptor species? Ecology and Evolution, 10(4), 1959–1970. 10.1002/ece3.6027 32128129 PMC7042680

[jane14173-bib-0035] Fischer, B. , Taborsky, B. , & Dieckmann, U. (2009). Unexpected patterns of plastic energy allocation in stochastic environments. The American Naturalist, 173(3), E108–E120. 10.1086/596536 PMC3359519 19196158

[jane14173-bib-0036] Fischer, B. , Taborsky, B. , & Kokko, H. (2011). How to balance the offspring quality–quantity tradeoff when environmental cues are unreliable. Oikos, 120(2), 258–270. 10.1111/j.1600-0706.2010.18642.x

[jane14173-bib-0037] Fung, Y. L. , Newman, K. , King, R. , & de Valpine, P. (2022). Building integral projection models with nonindependent vital rates. Ecology and Evolution, 12(3), e8682. 10.1002/ece3.8682 35342592 PMC8935301

[jane14173-bib-0038] Gabry, J. , & Češnovar, R. (2020). *cmdstanr: R Interface to* “*CmdStan*.” [Computer software].

[jane14173-bib-0039] Gascoigne, S. J. L. , Uwera Nalukwago, D. I. , & Barbosa, F. (2022). Larval density, sex, and allocation hierarchy affect life history trait covariances in a bean beetle. The American Naturalist, 199(2), 291–301. 10.1086/717639 35077283

[jane14173-bib-0040] Gebhardt, M. D. , & Stearns, S. C. (1988). Reaction norms for developmental time and weight at eclosion in Drosophila mercatorum. Journal of Evolutionary Biology, 1(4), 335–354. 10.1046/j.1420-9101.1988.1040335.x

[jane14173-bib-0041] Gelman, A. , & Rubin, D. B. (1992). Inference from iterative simulation using multiple sequences. Statistical Science, 7(4), 457–472. 10.1214/ss/1177011136

[jane14173-bib-0042] Gillespie, D. O. S. , Russell, A. F. , & Lummaa, V. (2008). When fecundity does not equal fitness: Evidence of an offspring quantity versus quality trade‐off in pre‐industrial humans. Proceedings of the Royal Society B: Biological Sciences, 275(1635), 713–722. 10.1098/rspb.2007.1000 PMC2366115 18211874

[jane14173-bib-0043] Hadfield, J. D. (2008). Estimating evolutionary parameters when viability selection is operating. Proceedings of the Royal Society B: Biological Sciences, 275(1635), 723–734. 10.1098/rspb.2007.1013 PMC2596846 18211873

[jane14173-bib-0044] Hamel, S. , Gaillard, J.‐M. , Douhard, M. , Festa‐Bianchet, M. , Pelletier, F. , & Yoccoz, N. G. (2018). Quantifying individual heterogeneity and its influence on life‐history trajectories: Different methods for different questions and contexts. Oikos, 127(5), 687–704. 10.1111/oik.04725

[jane14173-bib-0045] Healy, K. , Ezard, T. H. G. , Jones, O. R. , Salguero‐Gómez, R. , & Buckley, Y. M. (2019). Animal life history is shaped by the pace of life and the distribution of age‐specific mortality and reproduction. Nature Ecology & Evolution, 3(8), Article 8. 10.1038/s41559-019-0938-7 31285573

[jane14173-bib-0046] Holtmann, B. , Lagisz, M. , & Nakagawa, S. (2017). Metabolic rates, and not hormone levels, are a likely mediator of between‐individual differences in behaviour: A meta‐analysis. Functional Ecology, 31(3), 685–696. 10.1111/1365-2435.12779

[jane14173-bib-0047] Kain, M. P. , Bolker, B. M. , & McCoy, M. W. (2015). A practical guide and power analysis for GLMMs: Detecting among treatment variation in random effects. PeerJ, 3, e1226. 10.7717/peerj.1226 26401446 PMC4579019

[jane14173-bib-0048] Kendall, B. E. , Fox, G. A. , Fujiwara, M. , & Nogeire, T. M. (2011). Demographic heterogeneity, cohort selection, and population growth. Ecology, 92(10), 1985–1993. 10.1890/11-0079.1 22073789

[jane14173-bib-0049] Kengeri, S. S. , Maras, A. H. , Suckow, C. L. , Chiang, E. C. , & Waters, D. J. (2013). Exceptional longevity in female Rottweiler dogs is not encumbered by investment in reproduction. Age, 35(6), 2503–2513. 10.1007/s11357-013-9529-8 23584889 PMC3825016

[jane14173-bib-0050] Knops, J. M. H. , Koenig, W. D. , & Carmen, W. J. (2007). Negative correlation does not imply a tradeoff between growth and reproduction in California oaks. Proceedings of the National Academy of Sciences of the United States of America, 104(43), 16982–16985. 10.1073/pnas.0704251104 17940035 PMC2040414

[jane14173-bib-0051] Krajick, K. (2004). All downhill from here? Science, 303(5664), 1600–1602. 10.1126/science.303.5664.1600 15016975

[jane14173-bib-0052] Kroeger, S. B. , Blumstein, D. T. , Armitage, K. B. , Reid, J. M. , & Martin, J. G. A. (2020). Older mothers produce more successful daughters. Proceedings of the National Academy of Sciences of the United States of America, 117(9), 4809–4814. 10.1073/pnas.1908551117 32071200 PMC7060700

[jane14173-bib-0094] Kruuk, L. E. B. , Slate, J. , & Wilson, A. J. (2008). New answers for old questions: The evolutionary quantitative genetics of wild animal populations. Annual Review of Ecology, Evolution, and Systematics, 39(1), 525–548. 10.1146/annurev.ecolsys.39.110707.173542

[jane14173-bib-0053] Lack, D. (1947). The significance of clutch‐size. Ibis, 89(2), 302–352. 10.1111/j.1474-919X.1947.tb04155.x

[jane14173-bib-0054] Landes, J. , Henry, P.‐Y. , Hardy, I. , Perret, M. , & Pavard, S. (2019). Female reproduction bears no survival cost in captivity for gray mouse lemurs. Ecology and Evolution, 9(11), 6189–6198. 10.1002/ece3.5124 31236213 PMC6580269

[jane14173-bib-0055] Law, R. (1979). Optimal life histories under age‐specific predation. The American Naturalist, 114(3), 399–417. 10.1086/283488

[jane14173-bib-0056] Link, W. A. , & Eaton, M. J. (2012). On thinning of chains in MCMC. Methods in Ecology and Evolution, 3(1), 112–115. 10.1111/j.2041-210X.2011.00131.x

[jane14173-bib-0057] Martin, J. S. (2023). Covariance reaction norms: A flexible approach to estimating continuous environmental effects on quantitative genetic and phenotypic (co)variances. 10.32942/X2D89H

[jane14173-bib-0058] McElreath, R. (2020). Statistical rethinking: A Bayesian course with examples in R and Stan (2nd ed.). Chapman and Hall/CRC. 10.1201/9780429029608

[jane14173-bib-0059] Melcher, J. C. , Armitage, K. B. , & Porter, W. P. (1990). Thermal influences on the activity and energetics of yellow‐bellied marmots (*Marmota flaviventris*). Physiological Zoology, 63(4), 803–820. 10.1086/physzool.63.4.30158178

[jane14173-bib-0060] Messina, F. J. , & Fry, J. D. (2003). Environment‐dependent reversal of a life history trade‐off in the seed beetle *Callosobruchus maculatus* . Journal of Evolutionary Biology, 16(3), 501–509. 10.1046/j.1420-9101.2003.00535.x 14635850

[jane14173-bib-0061] Messina, F. J. , & Slade, A. F. (1999). Expression of a life‐history trade‐off in a seed beetle depends on environmental context. Physiological Entomology, 24(4), 358–363. 10.1046/j.1365-3032.1999.00151.x

[jane14173-bib-0062] Metcalf, C. J. E. (2016). Invisible trade‐offs: Van Noordwijk and de Jong and life‐history evolution. The American Naturalist, 187(4), iii–v. 10.1086/685487 27028085

[jane14173-bib-0063] Milner, J. M. , Elston, D. A. , & Albon, S. D. (1999). Estimating the contributions of population density and climatic fluctuations to interannual variation in survival of Soay sheep. Journal of Animal Ecology, 68(6), 1235–1247. 10.1046/j.1365-2656.1999.00366.x

[jane14173-bib-0065] Ozgul, A. , Childs, D. Z. , Oli, M. K. , Armitage, K. B. , Blumstein, D. T. , Olson, L. E. , Tuljapurkar, S. , & Coulson, T. (2010). Coupled dynamics of body mass and population growth in response to environmental change. Nature, 466(7305), Article 7305. 10.1038/nature09210 PMC5677226 20651690

[jane14173-bib-0068] Péron, G. , Gaillard, J.‐M. , Barbraud, C. , Bonenfant, C. , Charmantier, A. , Choquet, R. , Coulson, T. , Grosbois, V. , Loison, A. , Marzolin, G. , Owen‐Smith, N. , Pardo, D. , Plard, F. , Pradel, R. , Toïgo, C. , & Gimenez, O. (2016). Evidence of reduced individual heterogeneity in adult survival of long‐lived species. Evolution, 70(12), 2909–2914. 10.1111/evo.13098 27813056

[jane14173-bib-0066] Paniw, M. , Childs, D. Z. , Armitage, K. B. , Blumstein, D. T. , Martin, J. G. A. , Oli, M. K. , & Ozgul, A. (2020). Assessing seasonal demographic covariation to understand environmental‐change impacts on a hibernating mammal. Ecology Letters, 23(4), 588–597. 10.1111/ele.13459 31970918

[jane14173-bib-0093] Paterson, J. T. , Rotella, J. J. , Link, W. A. , & Garrott, R. (2018). Variation in the vital rates of an Antarctic marine predator: The role of individual heterogeneity. Ecology, 99(10), 2385–2396. 10.1002/ecy.2481 30277558

[jane14173-bib-0067] Pease, C. M. , & Bull, J. J. (1988). A critique of methods for measuring life history trade‐offs. Journal of Evolutionary Biology, 1(4), 293–303. 10.1046/j.1420-9101.1988.1040293.x

[jane14173-bib-0069] R Core Team . (2021). R: A language and environment for statistical computing. R Foundation for Statistical Computing. [Computer software].

[jane14173-bib-0070] Réale, D. , Reader, S. M. , Sol, D. , McDougall, P. T. , & Dingemanse, N. J. (2007). Integrating animal temperament within ecology and evolution. Biological Reviews, 82(2), 291–318. 10.1111/j.1469-185X.2007.00010.x 17437562

[jane14173-bib-0071] Regan, C. E. , Pemberton, J. M. , Pilkington, J. G. , & Smiseth, P. T. (2022). Having a better home range does not reduce the cost of reproduction in Soay sheep. Journal of Evolutionary Biology, 35(10), 1352–1362. 10.1111/jeb.14083 36063153 PMC9826142

[jane14173-bib-0072] Reznick, D. , Nunney, L. , & Tessier, A. (2000). Big houses, big cars, superfleas and the costs of reproduction. Trends in Ecology & Evolution, 15(10), 421–425. 10.1016/S0169-5347(00)01941-8 10998520

[jane14173-bib-0073] Ricklefs, R. E. , & Cadena, C. D. (2007). Lifespan is unrelated to investment in reproduction in populations of mammals and birds in captivity. Ecology Letters, 10(10), 867–872. 10.1111/j.1461-0248.2007.01085.x 17845285

[jane14173-bib-0074] Robinson, M. R. , & Beckerman, A. P. (2013). Quantifying multivariate plasticity: Genetic variation in resource acquisition drives plasticity in resource allocation to components of life history. Ecology Letters, 16(3), 281–290. 10.1111/ele.12047 23301600

[jane14173-bib-0075] Roff, D. A. (1995). The estimation of genetic correlations from phenotypic correlations: A test of Cheverud's conjecture. Heredity, 74(5), Article 5. 10.1038/hdy.1995.68

[jane14173-bib-0076] Salguero‐Gómez, R. , Jones, O. R. , Jongejans, E. , Blomberg, S. P. , Hodgson, D. J. , Mbeau‐Ache, C. , Zuidema, P. A. , de Kroon, H. , & Buckley, Y. M. (2016). Fast–slow continuum and reproductive strategies structure plant life‐history variation worldwide. Proceedings of the National Academy of Sciences of the United States of America, 113(1), 230–235. 10.1073/pnas.1506215112 26699477 PMC4711876

[jane14173-bib-0077] Searle, S. R. (1961). Phenotypic, genetic and environmental correlations. Biometrics, 17(3), 474–480. 10.2307/2527838

[jane14173-bib-0078] Sgrò, C. M. , & Hoffmann, A. A. (2004). Genetic correlations, tradeoffs and environmental variation. Heredity, 93(3), Article 3. 10.1038/sj.hdy.6800532 15280897

[jane14173-bib-0079] Simpson, E. H. (1951). The interpretation of interaction in contingency tables. Journal of the Royal Statistical Society: Series B: Methodological, 13(2), 238–241. 10.1111/j.2517-6161.1951.tb00088.x

[jane14173-bib-0080] Skrondal, A. , & Rabe‐Hesketh, S. (2007). Redundant overdispersion parameters in multilevel models for categorical responses. Journal of Educational and Behavioral Statistics, 32(4), 419–430. 10.3102/1076998607302629

[jane14173-bib-0081] Spigler, R. B. , & Woodard, A. J. (2019). Context‐dependency of resource allocation trade‐offs highlights constraints to the evolution of floral longevity in a monocarpic herb. New Phytologist, 221(4), 2298–2307. 10.1111/nph.15498 30256414

[jane14173-bib-0082] Stearns, S. , de Jong, G. , & Newman, B. (1991). The effects of phenotypic plasticity on genetic correlations. Trends in Ecology & Evolution, 6(4), 122–126. 10.1016/0169-5347(91)90090-K 21232440

[jane14173-bib-0083] Stearns, S. C. (1984). The effects of size and phylogeny on patterns of covariation in the life history traits of lizards and snakes. The American Naturalist, 123(1), 56–72. 10.1086/284186

[jane14173-bib-0084] Stearns, S. C. (1989). Trade‐offs in life‐history evolution. Functional Ecology, 3(3), 259–268. 10.2307/2389364

[jane14173-bib-0085] Stearns, S. C. (1992). The evolution of life histories. Oxford University Press.

[jane14173-bib-0086] Tavecchia, G. , Coulson, T. , Morgan, B. J. T. , Pemberton, J. M. , Pilkington, J. C. , Gulland, F. M. D. , & Clutton‐Brock, T. H. (2005). Predictors of reproductive cost in female Soay sheep. Journal of Animal Ecology, 74(2), 201–213. 10.1111/j.1365-2656.2005.00916.x

[jane14173-bib-0087] van Noordwijk, A. J. , & de Jong, G. (1986). Acquisition and allocation of resources: Their influence on variation in life history tactics. The American Naturalist, 128(1), 137–142. 10.1086/284547

[jane14173-bib-0088] van Tienderen, P. H. (1995). Life cycle trade‐offs in matrix population models. Ecology, 76(8), 2482–2489. 10.2307/2265822

[jane14173-bib-0089] Wells, C. P. , Barbier, R. , Nelson, S. , Kanaziz, R. , & Aubry, L. M. (2022). Life history consequences of climate change in hibernating mammals: A review. Ecography, 2022(6), e06056. 10.1111/ecog.06056

[jane14173-bib-0090] Williams, G. C. (1966). Natural selection, the costs of reproduction, and a refinement of Lack's principle. The American Naturalist, 100(916), 687–690. 10.1086/282461

[jane14173-bib-0091] Wilson, A. J. , & Nussey, D. H. (2010). What is individual quality? An evolutionary perspective. Trends in Ecology & Evolution, 25(4), 207–214. 10.1016/j.tree.2009.10.002 19897275

[jane14173-bib-0095] Wilson, A. J. , Réale, D. , Clements, M. N. , Morrissey, M. M. , Postma, E. , Walling, C. A. , Kruuk, L. E. B. , & Nussey, D. H. (2010). An ecologist's guide to the animal model. Journal of Animal Ecology, 79(1), 13–26. 10.1111/j.1365-2656.2009.01639.x 20409158

