# Supplementary material for: Detecting context dependence in the expression of life history trade‐offs
Source: J Anim Ecol. 2024 Sep 2;94(3):379–93. doi: 10.1111/1365-2656.14173 (PMC11880661; doi:10.1111/1365-2656.14173)
Supplement: Supplementary file 1 — Section S1. Fixed individual heterogeneity. Section S2. Posterior predictive checks. Section S3. Associations between the covariates and traits studied. [file JANE-94-379-s001.pdf]

Detecting context-dependence in the expression of life history tradeoffs

Supplementary materials

Section S1: fixed individual heterogeneity

The models presented in the main text do not include distinct parameters for fixed individual heterogeneity across environmental contexts, hence considering observations from the same individual but in different environmental contexts as independent. Here, we illustrate why this limitation is needed to correctly estimate context-dependent covariation. Using simulated data, we illustrate that it is not possible to estimate the among-individual variation across context, while at the same time estimating among- and within- individual variation within context. However, it is important to note that not accounting for fixed individual heterogeneity should not have any consequences regarding the accuracy of the estimation of the context-dependent correlations.

For this purpose, we simulate demographic data with a tradeoff between parental growth and fecundity, suitable for the non-repeated measures CRN (model of equation 2). We include a fixed heterogeneity component (context-independent individual random effect), as well as the context-dependent component (context-dependent individual random effect) to make the correlation vary across contexts. The data is similar to what is presented in the “validation on simulated datasets” section, with the addition of the fixed heterogeneity component. We then analyze this simulated dataset either with a model that estimates only the context-dependent covariation (model presented in the manuscript, equation 2), or a model that does include fixed

(context-invariant) individual random effects in addition to the context-dependent covariation (model of equation 2 with the addition of a context-independent individual random effect).

The figures for both scenarios are presented below, with Figure S1 highlighting that the inclusion of a fixed among-individual random effect in the model leads to an erroneous estimation of the context-dependent correlation. This is because the inclusion of this fixed among-individual random effect captures part of the variation from the context-dependent random effects, and therefore the context-dependent term will then only estimate deviations of individuals from the fixed heterogeneity term. However, Figure S2 highlights that not including a fixed among-individual random effect allows the model to properly recover the context-dependent correlation.

Our results here reflect a more general theoretical point about the biological interpretation of reaction norms. For any reaction norm model, there will not be a distinct component of fixed individual heterogeneity separated from the process of phenotypic plasticity shaping individual heterogeneity across environments. With simple linear reaction norms, empiricists often conceptualize the intercept of the model as reflecting a fixed, environmentally invariant component of the response, separate from the plastic effects described by reaction norm slopes. However, while this can be heuristically useful for some purposes, it is in a strict sense misleading, as the reaction norm intercept simply describes the variation expected when the environmental variable defining the slope is fixed to 0 (e.g., in the average environment for a mean-centered predictor or in the absence of an environmental exposure). Therefore, the value of the intercept is no more fixed than the expected value at any other position along the slope with respect to a fixed value of the environmental gradient. This thinking applies to the CRN and

any other reaction norm model. When sufficient data is available, individual random slopes could also be estimated, which can be used to directly quantify the degree to which individuals' rank order may shift across environments (Mitchell & Houslay, 2021). However, the depth of repeated sampling required to fit such models for present purposes is unlikely to be achieved by many currently existing datasets, motivating our CRN approach. Moreover, these random individual slopes will generally be of less interest for detecting demographic tradeoffs, as compared to the average shift in among-individual trait covariance across the population as determined by the fixed CRN slopes.

Taking a CRN approach to one's data thus requires taking seriously that there may not be any biologically meaningful sense in which there is a fixed level of individual heterogeneity irrespective of the environment (for traits that exhibit phenotypic plasticity). Rather, there is simply the amount of individual heterogeneity given a particular environment, prior to exposure to the environment, averaged across environments, and/or in the average environment. The parameters from the CRN can always be used to predict any such quantities of interest. For instance, applying the inverse link function to the intercept of the CRN (the first element of $\beta_{\boldsymbol{r}}$) will describe the expected trait correlation under the average environmental conditions.

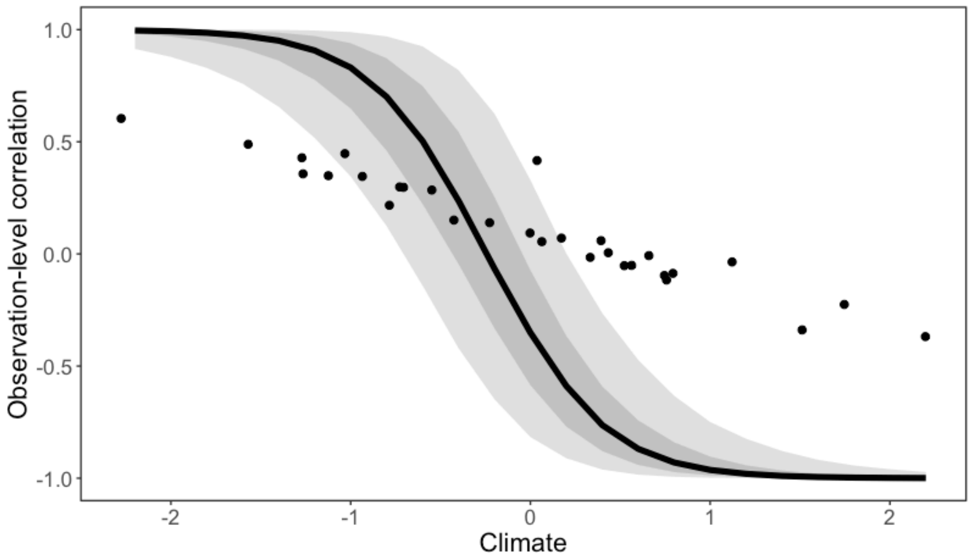

Figure S1: Estimated context-dependent correlation when a fixed individual-random effect is included. The regression line indicates the mean effect of climate on the correlation, while the shaded areas depict the 50% and 89% credible intervals predicted by the model. Each black dot represents the simulated observation-level correlation between both traits in a given year depending on climate. This highlights that the inclusion of a fixed individual random effect leads to a biased estimation of the context-dependent correlation.

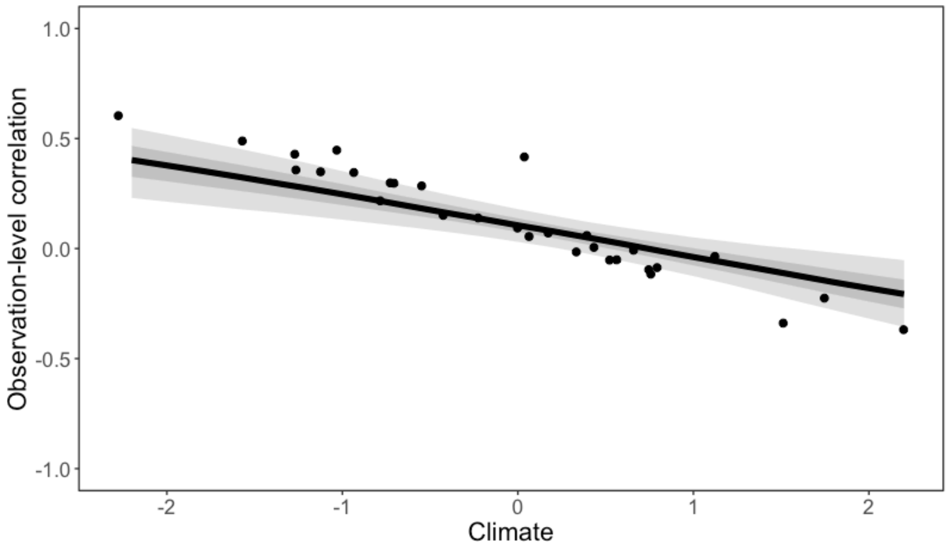

Figure S2: Estimated context-dependent correlation without the inclusion of a fixed individual-random effect in the model. The regression line indicates the mean effect of climate on the correlation, while the shaded areas depict the 50% and 89% credible intervals predicted by the model. Each black dot represents the simulated observation-level correlation between both traits in a given year depending on climate. This highlights that not including a fixed individual random effect leads to an appropriate estimation of the context-dependent correlation.

Section S2: posterior predictive checks

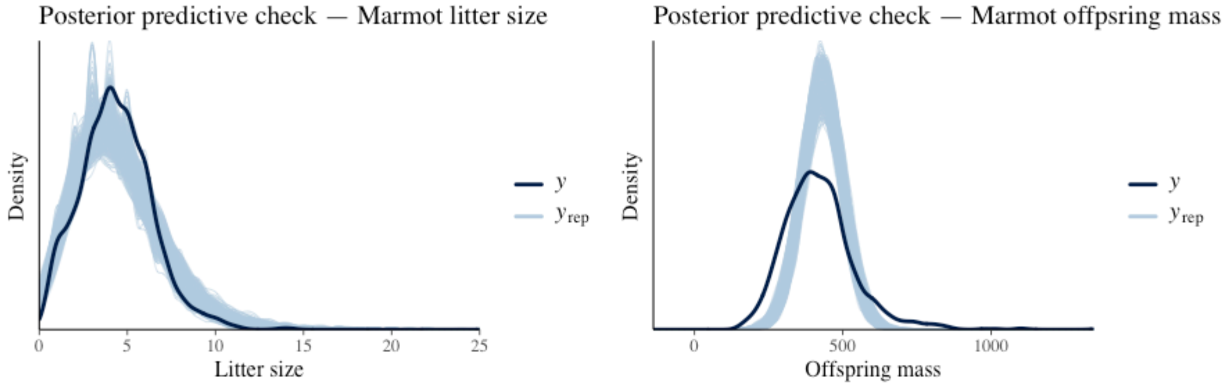

Figure S3: Posterior predictive checks showing the concordance between the distribution of the data (y) and the distribution of data generated under the statistical model (y_{rep}), for litter size (left panel) and offspring mass (right panel). This highlights a good fit for the litter size model. It also highlights that there is a slight overdispersion in offspring mass that is not accounted for by the model.

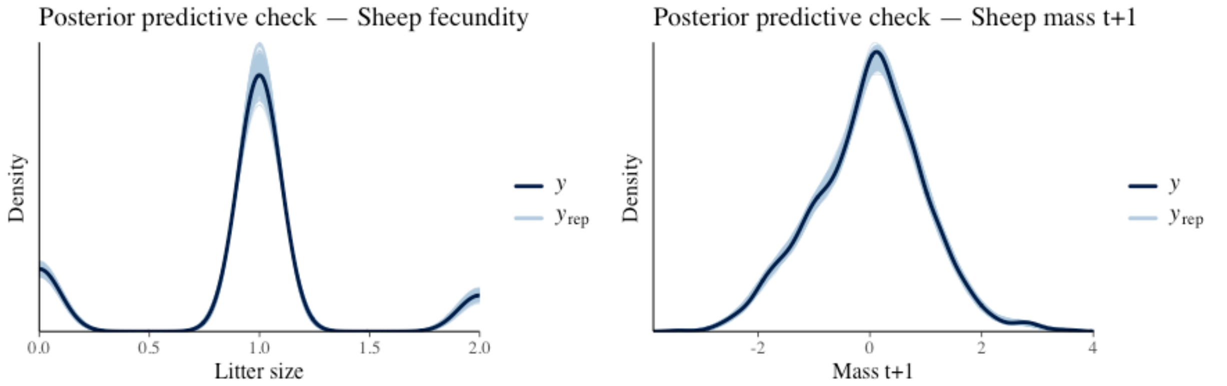

Figure S4: Posterior predictive checks showing the concordance between the distribution of the data (y) and the distribution of data generated under the statistical model (y_{rep}), for number of offspring (left panel) and ewe's mass in the following summer (right panel). This highlights a good fit for both the litter size and mass models.

Section S3: associations between the covariates and traits studied

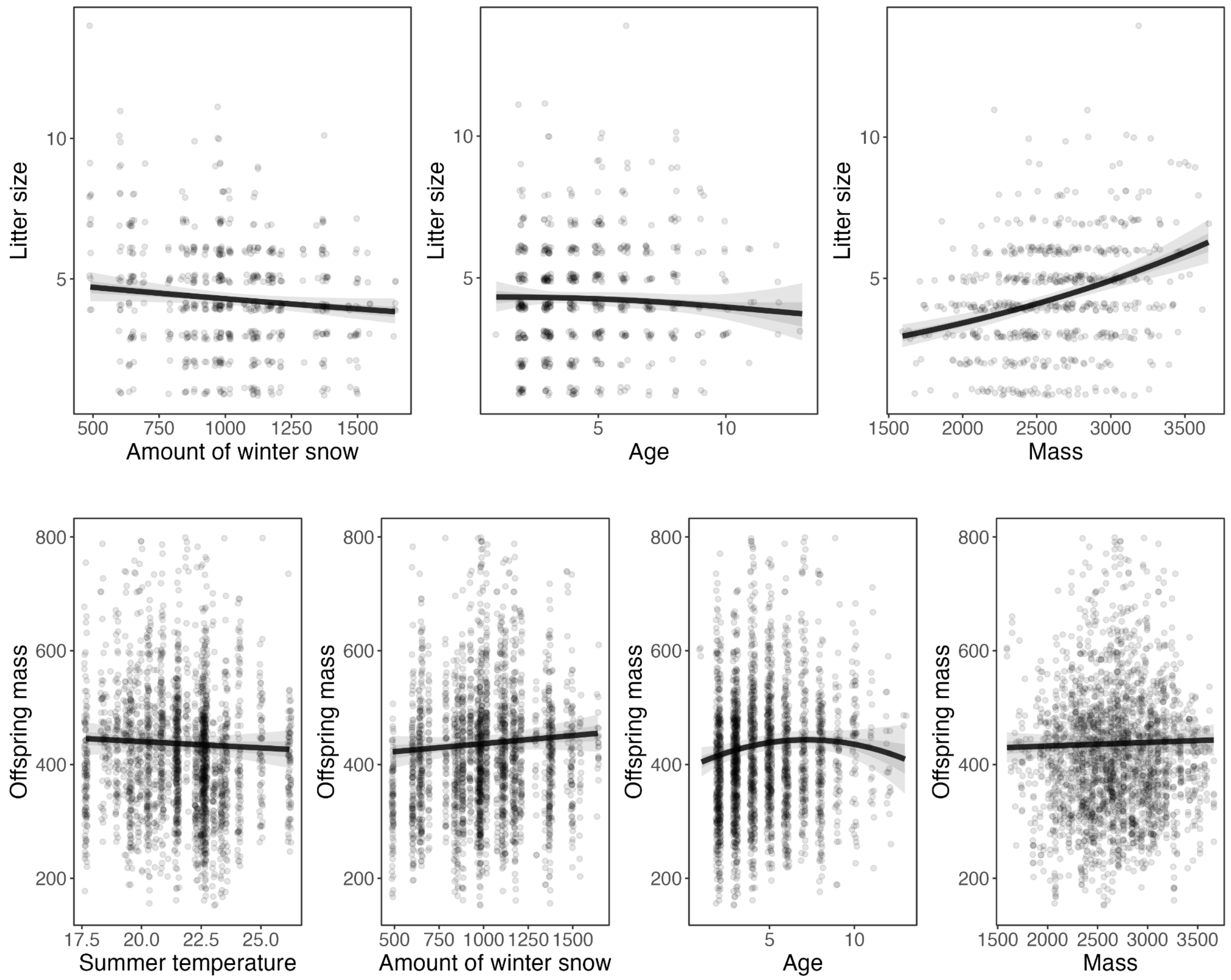

Figure S5: Top row: Association estimated by the model between the amount of winter snow, age, and mass (panels from left to right) with litter size. Bottom row: Association estimated by the model between summer temperature, the amount of winter snow, age, and mass (panels from left to right) with offspring mass.

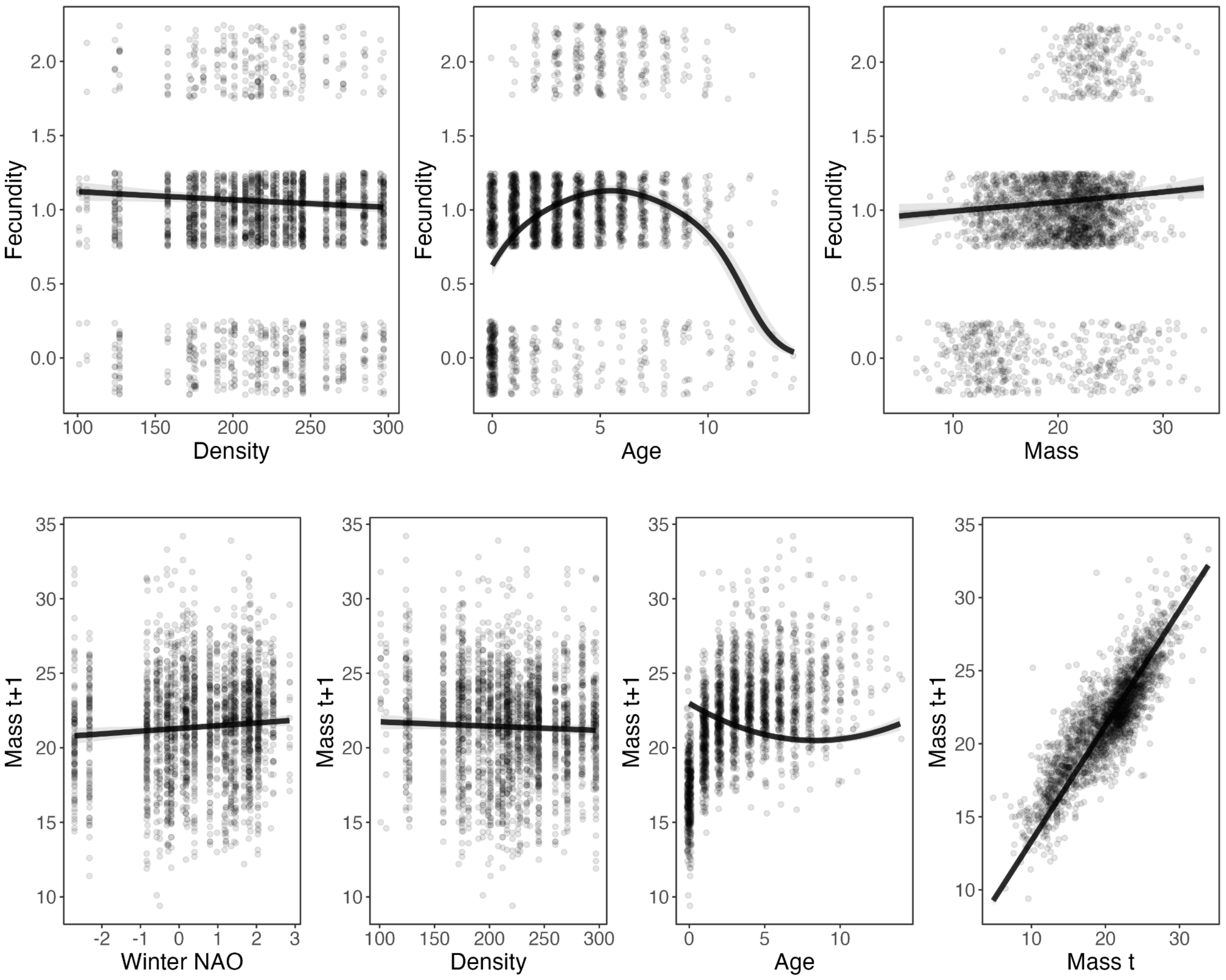

Figure S6: Top row: Association estimated by the model between population density, age, and mass (panels from left to right) with fecundity. Bottom row: Association estimated by the model between winter NAO, population density, age, and mass (panels from left to right) with mass at t+1.
